# A Pair of Fluorescent Probes Enabling Precise Diagnosis of Liver Cancer by Complementary Imaging

**DOI:** 10.1021/acscentsci.4c01822

**Published:** 2024-12-16

**Authors:** Min Gao, Sun Hyeok Lee, Haw-Young Kwon, Larissa Miasiro Ciaramicoli, Eunsol Jo, Young Hyun Yu, Fengming Li, Beomsue Kim, Kyungtae Hong, Jun-Seok Lee, Namhui Kim, Yoojin Oh, Chun Young Im, Chris Soon Heng Tan, Hyung-Ho Ha, Young-Tae Chang

**Affiliations:** [a] School of Chemistry and Chemical Engineering, Linyi University, Linyi 276005, P. R. China; [b] School of Interdisciplinary Bioscience and Bioengineering, Pohang University of Science and Technology (POSTECH), Pohang 37673, Republic of Korea; [c] Department of Chemistry, Pohang University of Science and Technology (POSTECH), Pohang 37673, Republic of Korea; [d] College of Pharmacy and Research Institute of Life and Pharmaceutical Sciences, Sunchon National University, Sunchon 57922, Republic of Korea; [e] Department of Chemistry, Southern University of Science and Technology, Shenzhen 518055, P. R. China; [f] Neural Circuits Research Group, Korea Brain Research Institute, Daegu 41062, Republic of Korea; [g] Bio-Med Program, KIST-School UST, Biomedical Research Division, Korea Institute of Science and Technology, Seoul 02792, Republic of Korea; [h] Department of Pharmacology, Korea University, Seoul 02841, Republic of Korea; [i] New Drug Development Center, Daegu-Gyeongbuk Medical Innovation Foundation(K-MEDIhub), Daegu 41061, Republic of Korea

## Abstract

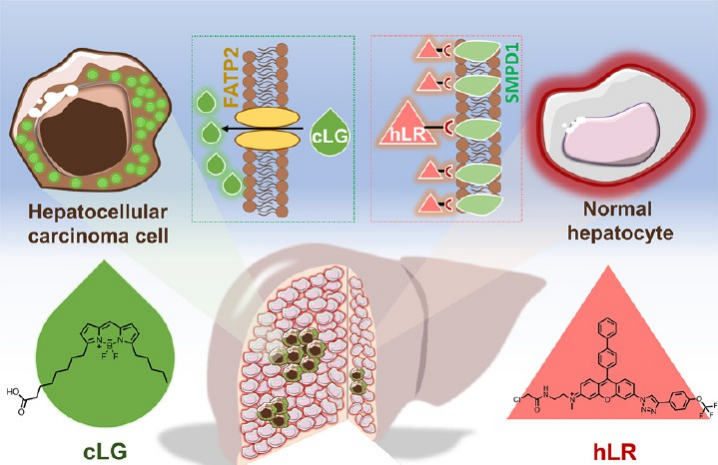

Hepatocellular carcinoma (HCC) is by far the predominant malignant liver cancer, with both high morbidity and mortality. Early diagnosis and surgical resections are imperative for improving the survival of HCC patients. However, limited by clinical diagnosis methods, it is difficult to accurately distinguish tumor tissue and its boundaries in the early stages of cancer. Herein, we report two fluorescent probes, **cLG** and **hLR**, for the detection of cancer and healthy cells, respectively, enabling the precise diagnosis of liver cancer by providing complementary imaging. These two fluorescent probes could selectively stain the target cells in the liver tissue imaging, which is confirmed by H&E and antibody staining. Moreover, for the first time, the cancerous area and healthy area are clearly identified by the cocktail of these two probes, suggesting its potential to be used in fluorescence-guided surgery. Finally, we identify transporter SLC27A2 as the gating target of **cLG** through a systematic transporter screen using a CRISPR activation library. SMPD1 was identified as the target of **hLR** through a thermal proteome profiling. Therefore, the development of these two highly specific probes offers complementary imaging and provides a unique diagnostic tool for cancer disease, even for fluorescence-guided surgery.

## Abstract 2

cLG and hLR are presented to detect cancerous and healthy cells, respectively. This pair of fluorescent probes can accurately imaging liver cancer by providing dual-color imaging.

## Introduction

Hepatocellular carcinoma (HCC) is a primary malignancy of the liver that is associated with an increasing incidence and mortality rate. With annually reported cases, it is the sixth most common malignancy worldwide, and second only to lung cancer in terms of mortality.^1,2^ Early diagnosis and early surgical resections are imperative to improve the survival of HCC patients. In medicine, Alpha-fetoprotein (AFP), a specific glycoprotein produced primarily by the fetal liver, has been the most practical and widely used serum biomarker for early HCC diagnosis. However, its sensitivity and specificity vary significantly from 40%–65% and 76%–96%, respectively.^3,4^ The clinical diagnostic accuracy of AFP is unsatisfactory due to the wide variation in its sensitivity and specificity observed making elevated AFP nonspecificity, especially in the early stages of HCC.^5^ Additionally, magnetic resonance imaging (MRI), computed tomography (CT), positron emission tomography (PET), and ultrasound are frequently applied to detect and diagnose HCC.^6^ Among all the diagnostic imaging modalities, MRI is more sensitive; however, it also bears some inherent limitations, such as its high cost, large footprint, long imaging times, and false positive contrast enhancement.^7^ Therefore, this has opened up potential in the research field to develop a new approach to achieve early diagnosis and better prognosis of HCC.

Among the emerging imaging modalities, fluorescence imaging has become a powerful method for the accurate diagnosis of cancer due to its unique advantages, including good sensitivity and selectivity, *in situ* and/or real-time detection, high spatiotemporal resolution, and noninvasive monitoring ability in living systems.^8^ Over the past decade, we have contributed to the field of small-molecule fluorescent probes via the Diversity-Oriented Fluorescence Library Approach (DOFLA) and have reported about 20 probes capable of live cell distinction by combinatorial screening.^9^ Depending on their different targets, the staining mechanism of the probe for the specific cells was elucidated as hold-oriented live cell differentiation (HOLD) and gate-oriented live cell differentiation (GOLD).^10,11^

For an accurate diagnosis of cancer, a high signal-to-background ratio (STBR) is required to distinguish cancerous tissue from normal tissue, enabling precise resection of HCC while minimizing unnecessary damage to adjacent normal liver tissue.^12^ Furthermore, STBR is particularly important for HCC because the liver is a metabolic organ. However, some studies have reported that using one fluorescent probe can distinguish liver cancer from healthy cells but cannot achieve high STBR.^13−20^ Therefore, to overcome this problem, developing cancer cell-specific and healthy cell-specific contrast agents is the preferred method to obtain a high STBR.

In this study, we presented a pair of fluorescent probes targeting cancer and healthy cells, respectively, based on high throughput screening with thousands of compounds. These two probes were successfully applied for tissue imaging and clearly distinguished the cancerous and healthy area. Moreover, for the first time, the cancerous area and healthy area were well identified with the combination of these two probes, enabling accurate cancer diagnosis and providing potential for fluorescence-guided surgery. Finally, the staining mechanism of cLG was identified as the FATP2 transporter through a systematic transporter screen using a CRISPR activation library. SMPD1 was identified as the target of hLR by thermal proteome profiling.

## Results and Discussion

### Discovery of Fluorescent Probes for Healthy and Cancer Cells

**Figure 1:**
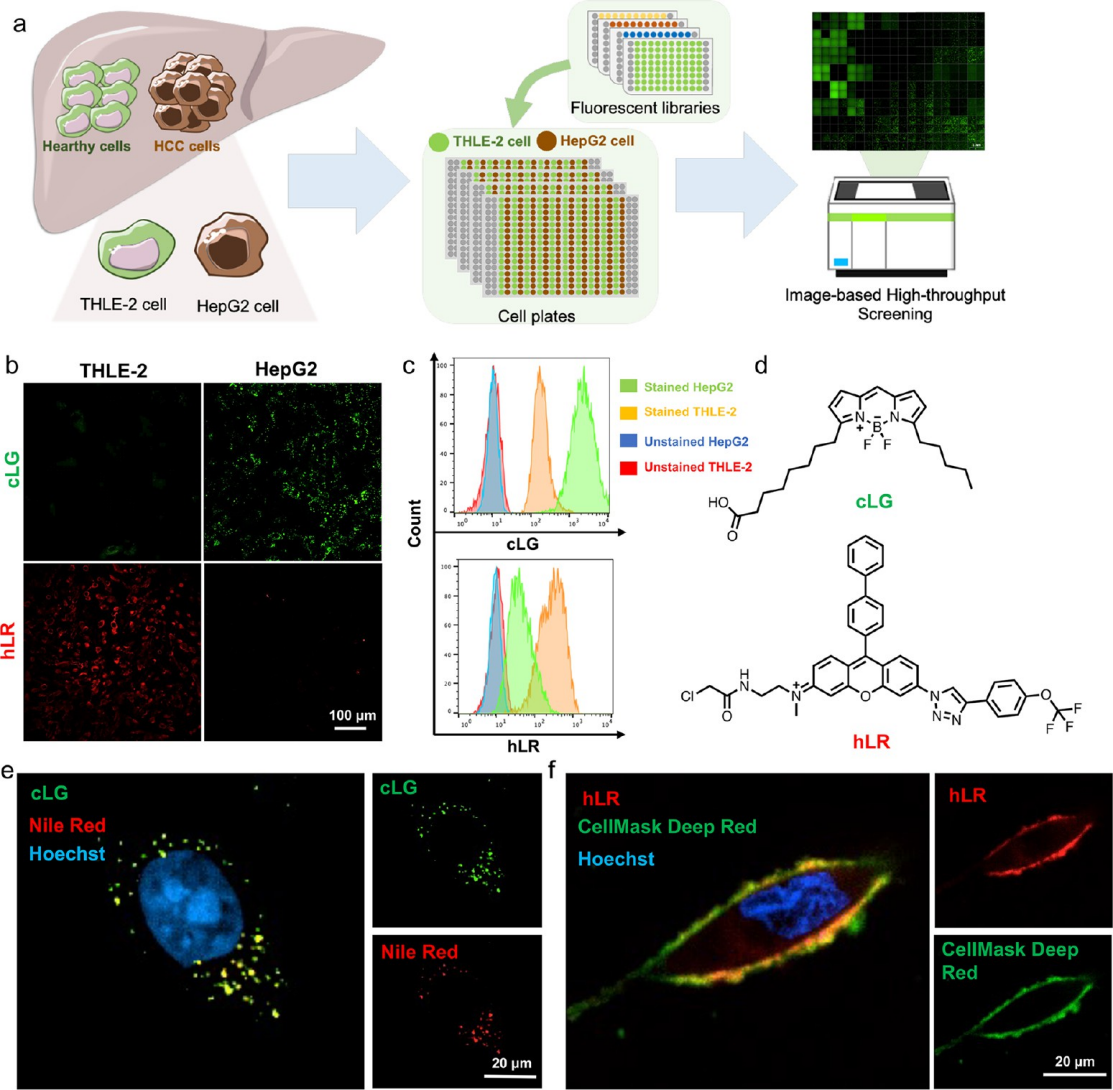
Discovery of fluorescent probe for HepG2 and THLE-2 cells. (a) Screening format. (b, c) The fluorescent image and flow cytometry of cLG and hLR in THLE-2 and HepG2 cells. The cells were treated with cLG (1 μM) or hLR (1 μM) for 1 h. (d) The structure of cLG and hLR. (e) and (f) The localization of cLG and hLR in HepG2 cells and THLE-2 cells, respectively. Concordant results were obtained from three independent experiments.

The limited STBR of fluorescent probes can hinder their ability to accurately monitor liver tumors, resulting in incomprehensive information on liver cancer. Developing two fluorescent probes specific for cancer and healthy cells is an innovative method to achieve a high STBR. Therefore, we selected THLE-2 and HepG2 cells to construct the screening platform. The THLE-2 cell is derived from healthy hepatocytes, while the HepG2 cell, the most used HCC cell line, closely resembles HCC tumors based on their gene expression.^21−23^ Over 8000 fluorescent library compounds were collected and tested side by side in THLE-2 and HepG2 cells using a high-throughput imaging microscope (Figure 1a). Compounds with higher fluorescent staining in one cell type compared with the other cell type were selected as primary hits. After 3 repeated experiments, **cLG** (**c**ancerous **L**iver **G**reen) and **hLR** (**h**ealthy **L**iver **R**ed) were chosen as the hits for HepG2 cells and THLE-2 cells, respectively, with the best contrast and stability (Figure 1b–d and Figure S25 and S26). **cLG** selectively stained the HepG2 cells, but not the THLE-2 cells. The **hLR** was the opposite (Figure 1b). The Z-factor for **cLG** is 0.60 and for **hLR** is 0.63. The specificity and reliability of these two probes for the target cells were further confirmed by flow cytometry (Figure 1c). In addition, multiple hepatocellular carcinoma cells were used to verify the selective staining of **cLG** and **hLR** (Figure S28).

Then, the cellular localization of the two probes was investigated prior to verification of the selective mechanism. In the subcellular level, **cLG** was localized in the lipid droplets, as the signal was well matched with commercial lipid droplet dye Nile Red (Figure 1e), possibly because the structure of **cLG** was fatty-acid-mimicking and highly lipophilic, which contributed to its localization in the lipid droplet. The **hLR** signal was overlapped with the cell membrane dye, suggesting it is bound to the cell membrane (Figure 1f and Figure S29).

### Cancer Cell Recognition in Cancerous Liver Section Using cLG

**Figure 2:**
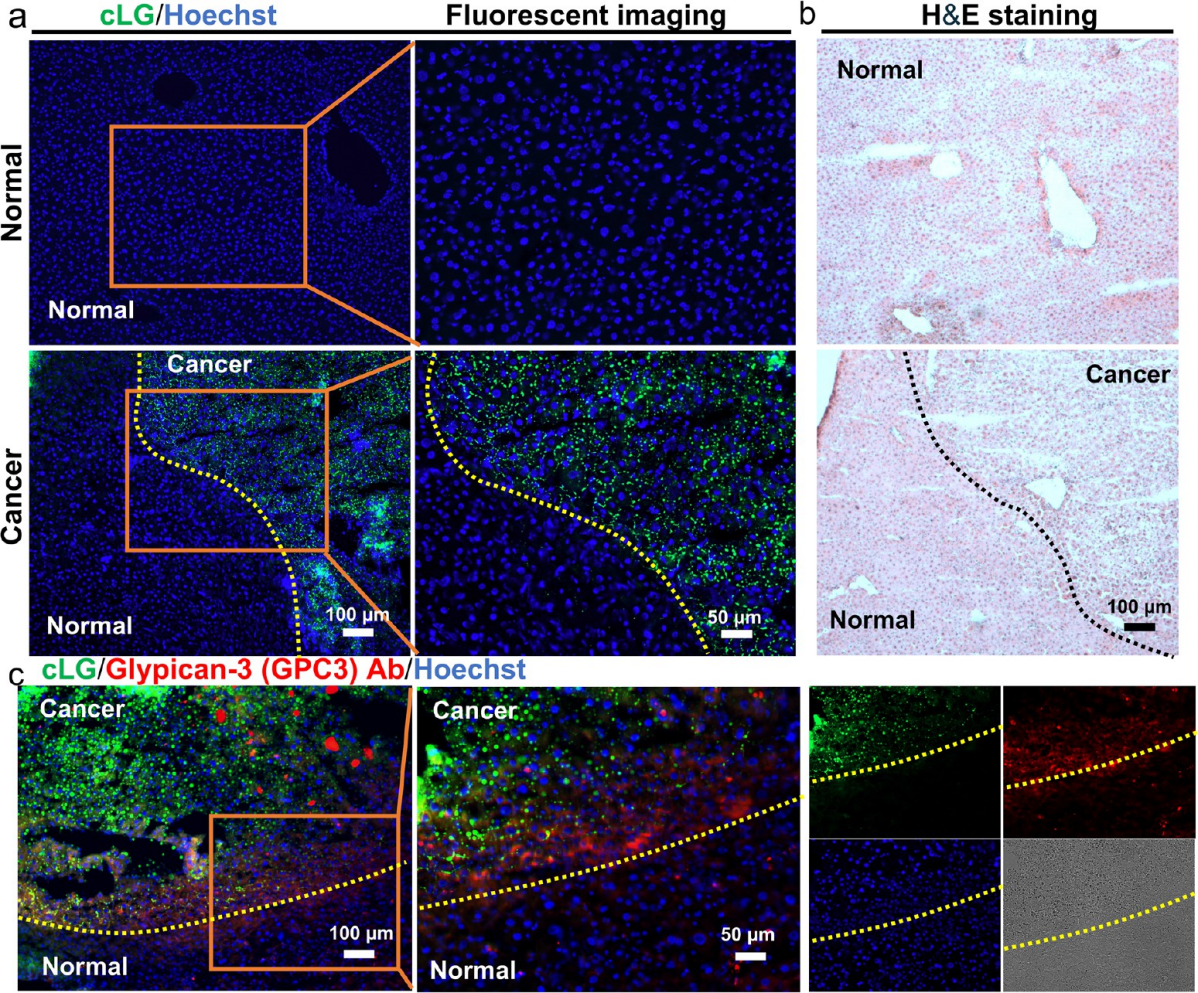
Cancer cell recognition in cancerous liver tissue using **cLG**. (a) Liver tissue imaging of **cLG**. The liver tissues were incubated with **cLG** (5 μM) for 30 min. (b) H&E staining of the liver tissues. (c) Colocalization of anti-GPC and **cLG** in the liver tissue. Concordant results were obtained from three independent experiments.

Motivated by the performance of **cLG** in liver cancer cells, we attempted to investigate its applicability in the field of cancer diagnosis. First, a mouse model of DEN-induced HCC was established to obtain the cancerous liver (Figure S30).^24^ Normal mice were set as controls. **cLG** was then applied to the liver tissue to assess whether it could light up cancer cells in the cancerous liver tissue. As expected, **cLG** showed faint fluorescence in the control liver section. In the cancerous liver section, **cLG** showed a clear distinction between cancer cells and normal cells (Figure 2a). And this distinction was very similar to H&E staining (Figure 2b).

In addition, to further confirm that the **cLG** labeled the cancer cells in the cancerous liver section, antiglypican-3 was applied and costained with **cLG**. Glypican 3 (GPC3), a glycosylphosphatidylinositol-anchored heparan sulfate proteoglycan, is the biomarker for hepatocellular carcinoma tissues.^25^ The fluorescence signal of **cLG** was strongly detected in the tumor area, which generally overlapped with antiglypican-3 staining (Figure 2c). Overall, **cLG** could accurately labeled the cancer cells in the tissue section, which could be confirmed by H&E and antibody staining.

### Healthy Liver Cell Recognition in Cancerous Liver Section Using hLR

**Figure 3:**
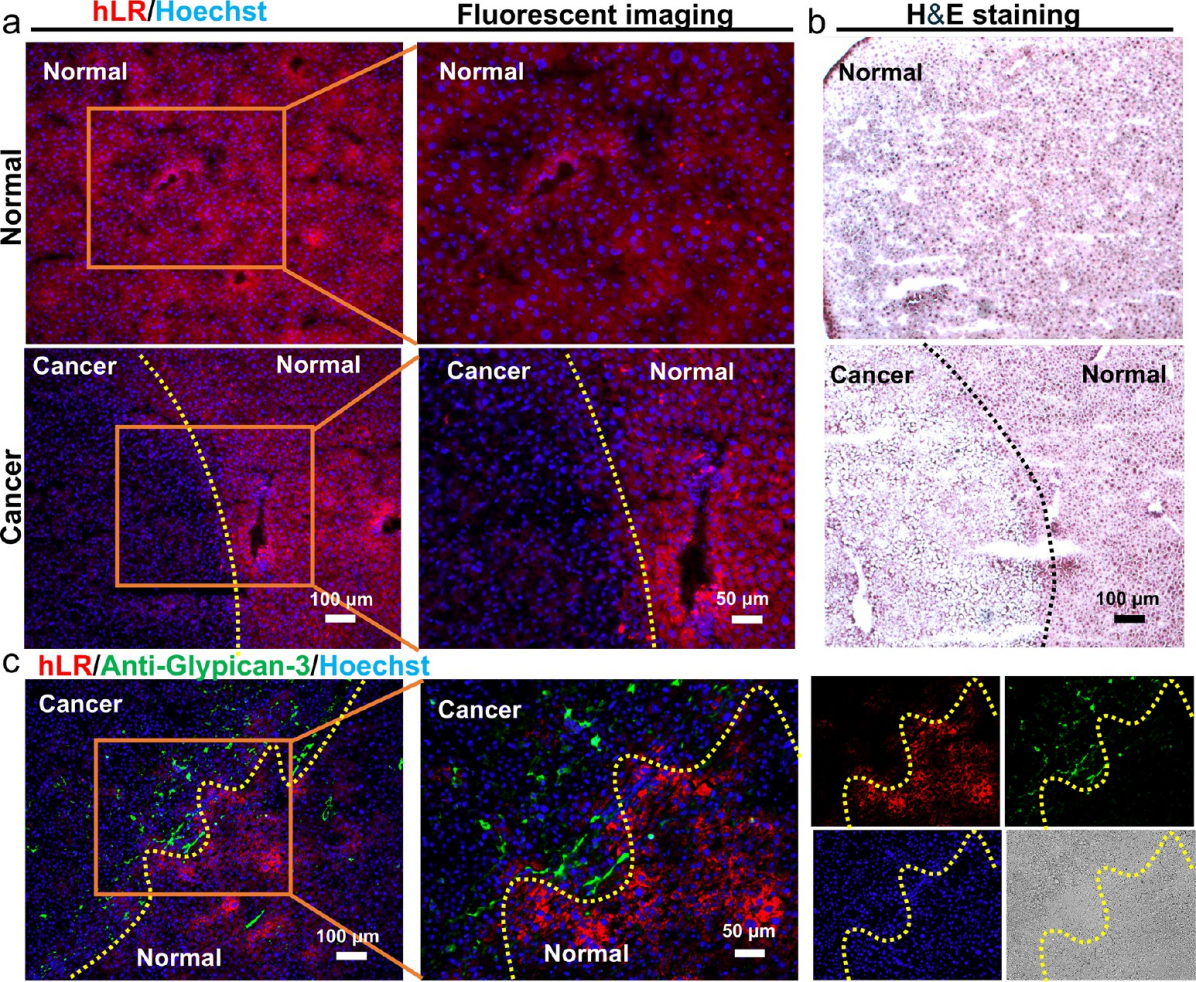
Healthy liver cell recognition in the cancerous liver section using **hLR**. (a) The liver tissues were incubated with **hLR** (10 μM) for 30 min. (b) H&E staining of the liver tissues. (c) Colocalization of anti-GPC and **hLR** in the liver tissue. Concordant results were obtained from three independent experiments.

Considering that **hLR** was selective for healthy hepatocytes, imaging of the liver tissue section was also performed to explore its application. Similarly, cancerous liver tissues were also obtained from the DEN-induced HCC mice. Normal liver tissues were from 8.5-month-old male mice. Contrary to **cLG**, **hLR** showed higher fluorescent intensity in the normal liver tissue section, while, in the cancerous liver tissue section, the signal was not observed in the cancerous region which was confirmed with H&E staining (Figure 3a,b). The distinction between cancer cells and healthy cells was matched by the fluorescence imaging and H&E staining (Figure 3a,b).

Similarly, the antiglypican-3 was also applied in the cancerous liver section to further confirm that **hLR** labeled the healthy liver cells. **hLR** did not show any signal in the cancerous region where antiglypican 3 stained (Figure 3c). Altogether, **hLR** could selectively stain the healthy liver cells in the tissue section, which could be confirmed by H&E and antibody staining.

### Costaining of cLG and hLR in the Cancerous Liver Tissue

**Figure 4:**
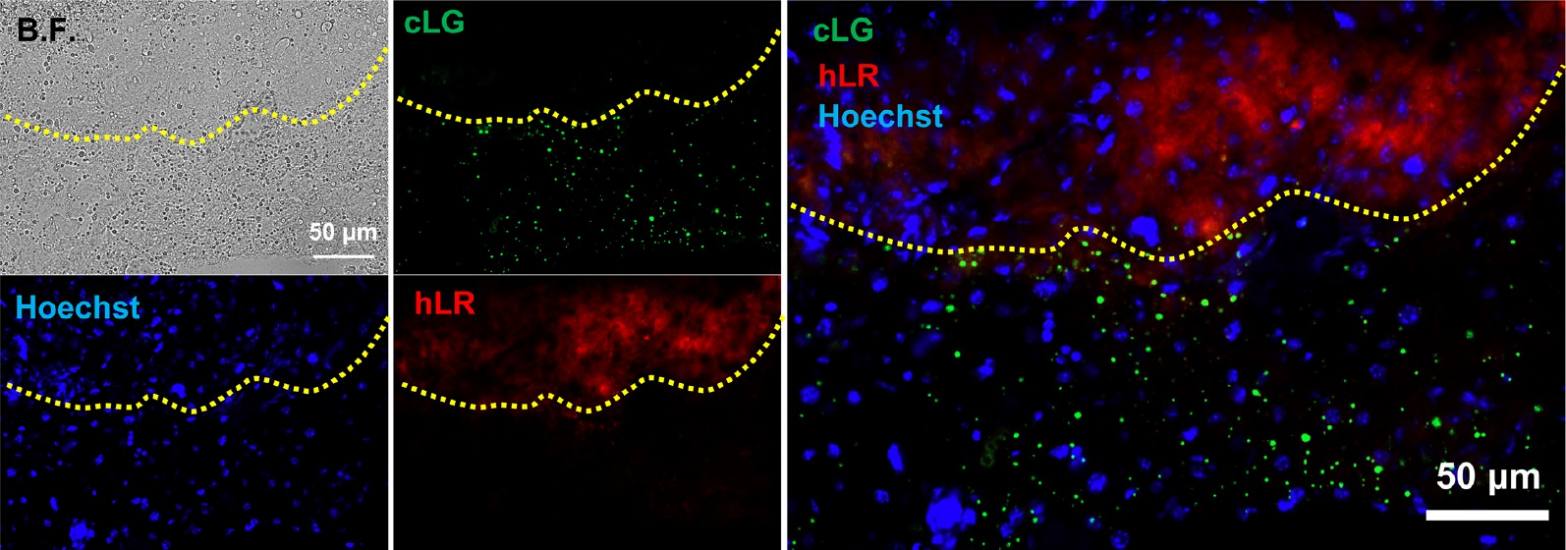
Costaining of **cLG** and **hLR** in cancerous liver tissue. The cocktail was made with **hLR** (10 μM) and **cLG** (5 μM). The tissue section was incubated with cocktail for 30 min. Tissue thickness is 15 μm. Concordant results were obtained from three independent experiments.

Surgical resection of cancer remains an important treatment modality. The presence of residual tumor cells after resection is considered as a strong predictor of tumor recurrence if resection is minor; otherwise, healthy tissue may be harmed.^26^ Since **cLG** and **hLR** stained the different sides of the coin, this may increase STBR for accurate diagnosis of cancer. To evaluate whether it could provide a high contrast between cancer cells and healthy cells, we made a cocktail of these two dyes and applied it to sections of cancerous liver tissue. With this cocktail, the **cLG** signal and **hLR** signal did not overlap, indicating that cancer cells were well distinguished from healthy cells (Figure 4). Therefore, the combination of these two probes showed high-contrast imaging, offering the potential for fluorescence-guided surgery.

### *Ex Vivo* Imaging of cLG and hLR

If **cLG** and **hLR** could be applied to *in vivo* imaging rather than being limited to tissue sections, a more accurate diagnosis of hepatocellular carcinoma would be possible. In this study, 100 μM **cLG**/**hLR** in PBS (100 μL) was intravenously injected into mice. To see the organs clearly, then the heart, liver, spleen, lung, and kidney were isolated and imaged *ex vivo* after 1 h. In the liver cancer model, the fluorescence of **cLG** appeared exactly in the tumor areas of the liver. While no fluorescence was observed in the liver of control mice (Figure S31a). Therefore, the selective imaging of **cLG** makes it a promising tool for fluorescence-guided surgery. However, **hLR** showed no selectivity between normal and tumor areas in the liver (Figure S31b). Additionally, the kidneys in both groups showed fluorescence due to the metabolism of **cLG** and **hLR**.

### Staining Mechanism of cLG

**Figure 5:**
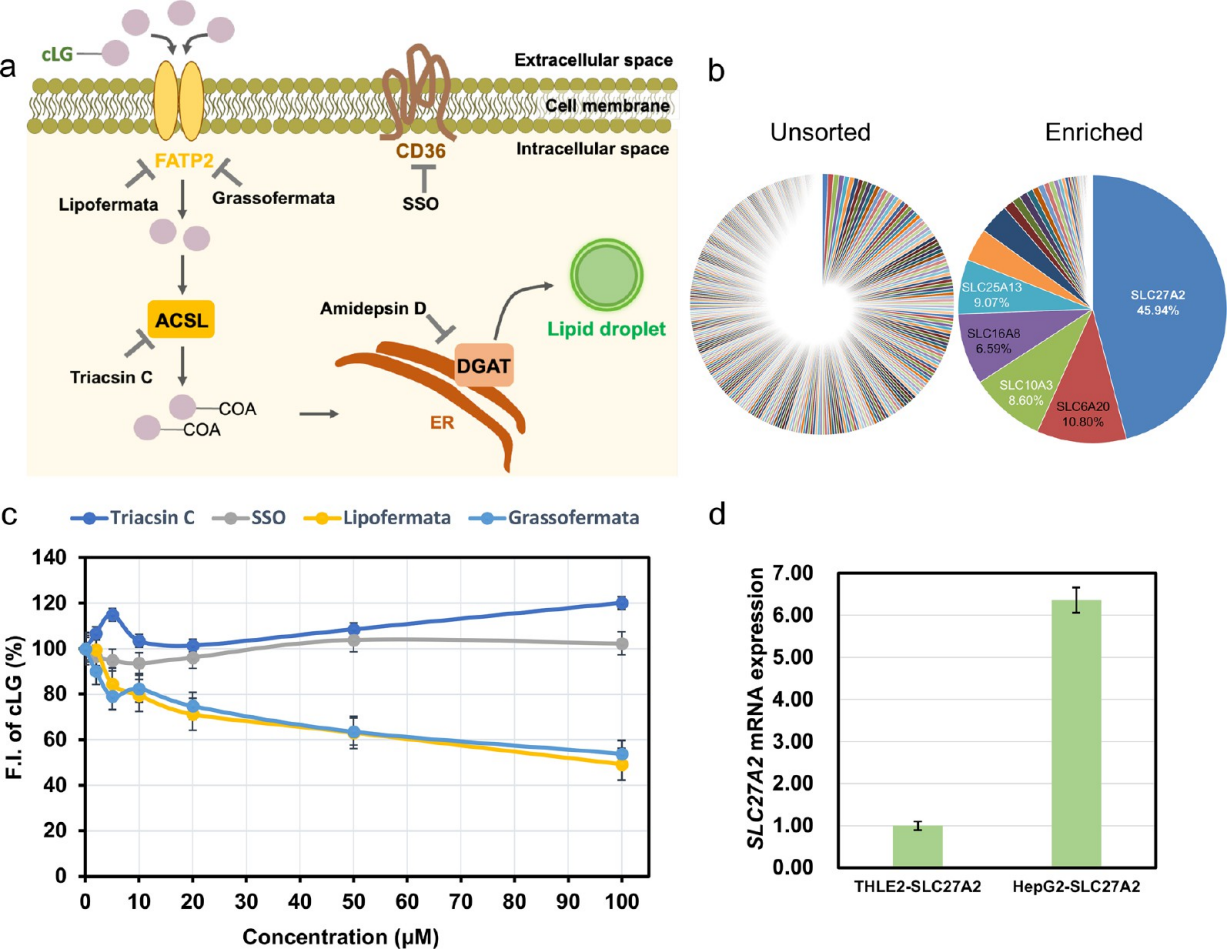
Staining mechanism of **cLG**. (a) The proposed staining mechanism of **cLG** on HepG2 cells. (b) NGS counts of the top 1000 highly enriched sgRNAs in the unsorted and the 4-round enriched populations. The percentages of each sgRNA count to the total count are represented as a pie chart. (c) The effect of inhibitors on the uptake of **cLG**. The HepG2 cells were treated with inhibitors for 30 min and then incubated with **cLG** (0.2 μM) for 1 h. (d) The SLC27A2 mRNA expression between THLE-2 and HepG2 cells. The data are presented as the mean ± standard deviation (SD). A minimum of three experiments per experiment was run. The error bars are derived from the SD. Concordant results were obtained from three independent experiments.

To identify the selective staining mechanism of fatty acid (FA)-mimicked **cLG**, we sought to explore the effect of FA transporters on the staining of **cLG**. The free FA is transported to the intracellular system mainly through CD36 and solute carrier family 27 (SLC27 or FA transporter proteins; FATPs). After uptake into the cells, FA is converted to acyl-CoA with the help of synthetases (ACSL), and then acyl CoA:diacylglycerol acyltransferase (DGAT) on the endoplasmic reticulum (ER) synthesizes the acyl-CoA for triacylglycerol (TG) for LD formation (Figure 5a).^27,28^

SLC27 contains six subtypes, namely, SLC27A1-SLC27A6, all of which should be considered as the **cLG** potential target protein. SLC-CRISPRa (CRISPR activation) and CRISPRi (CRISPR inhibition) systems are methods to screen SLC transporters for target identification, which has been successfully exploited to identify targets for CDg16 and CDyB.^29,30^ To generate single SLC enhanced cellular pools, 10 single guide RNAs (sgRNAs) targeting each of 380 protein-encoded SLC genes selected from NCBI Gene (http://www.ncbi.nlm.nih.gov/gene) were transfected to dCas9-VPR Hela, to create SLC-CRISPRa pools with stable overexpression of 3800 sgRNA.^31,32^ Therefore, **cLG** was then applied to the SLC-CRISPRa system for the schematic screening process to identify SLC transporters for the **cLG** import. After 4 round-enrichments of the top 5% brightest cell population, the sorted bright cell population showed stronger **cLG** staining than the unsorted cell population (Figure S32). Analyzing genomic DNA of the sorted SLC-CRISPRa population using next generation sequencing (NGS), it was revealed that the highest enriched sequence targeted SLC27A2, while sequences targeting SLC6A20, SLC25A13, SLC10A3, and SLC16A8 were at relatively low levels (Figure 5b). Among these candidate targets, SLC27A2 belongs to the SLC27 family expected as a promising target for **cLG** since it is known for FA transport. Other transporters are not involved in FA transport. Subsequently, the inhibitors for SLC27A2 were directly applied to HepG2 cells to evaluate the **cLG** uptake. After the addition of lipofermata or grassofermata, the fluorescent intensity of **cLG** was significantly reduced by up to 50%, indicating that SLC27A2 (FATP2) contributed to the **cLG** staining in the HepG2 cells (Figure 5c). Meanwhile, SLC27A2 (FATP2) was highly expressed in HepG2 compared to THLE-2 cells, providing additional evidence to imply that SLC27A2 could be the target for **cLG** (Figure 5d).

In addition, the SSO (CD36 inhibitor) and Triacsin C (ACSL inhibitor), respectively, were added to check the effect on **cLG** staining. However, a significant fluorescence decrease of **cLG** was not observed, indicating CD36 and ACSL did not affect the **cLG** uptake (Figure 5c).

### Staining Mechanism of hLR

**Figure 6:**
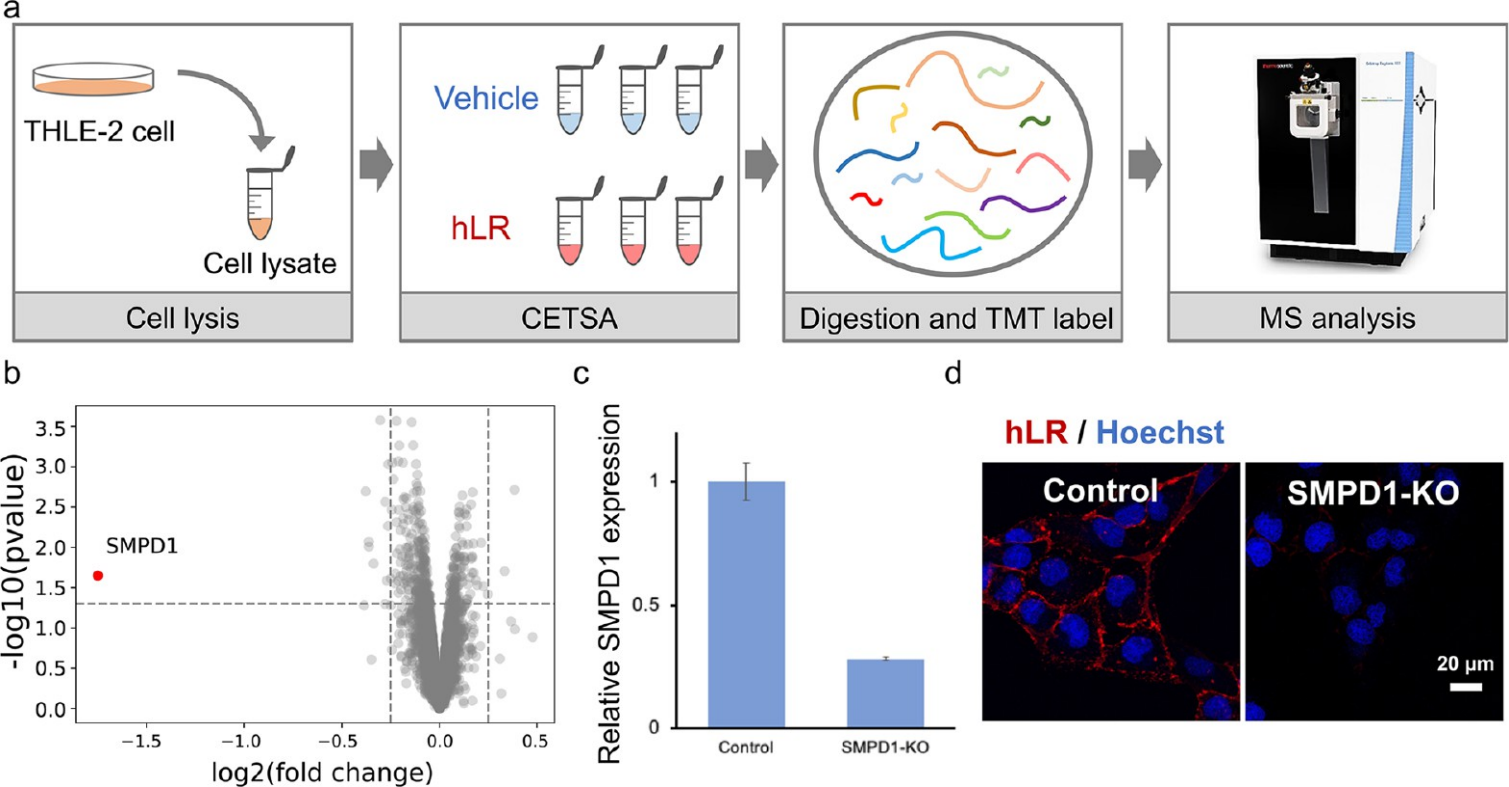
Staining mechanism of **hLR**. (a) Workflow of TPP that integrated preparation of cell lysates, CETSA, digestion, and TMT label and MS analysis. (b) Volcano plot visualization reveals SMPD1 is the top hit based on statistical significance and fold change. (c) SMPD1 gene level from control and SMPD1 KO THLE-2 cells. Data were pooled from three individual experiments. Data are analyzed with three samples over three independent experiments (*n* = 3). (d) Fluorescence intensity of control and SMPD1 KO THLE-2 cells. hLR (1 μM) was added after 30 min. All of the images were acquired at ×20 magnification. Data are presented as mean values ± SD (*n* = 3). All the error bars represent standard deviation from three independent measurements.

The localization of **hLR** is the plasma membrane (Figure 1f), leading us to hypothesize that it may bind to a cell surface protein. Plasma membrane proteins play multiple functions in intercellular recognition, signaling, and nutrient transport. In this study, thermal proteome profiling (TPP) was used for target protein identification. This technique combines cellular thermal shift analysis (CETSA) with quantitative protein mass spectrometry (MS) and is often used to identify interactions of drugs and chemicals with endogenous proteins. To identify the target, the THLE-2 cell lysate was treated with hLR, followed by CETSA and using SISPROT for protein digestion with manual fractionation of TMT-labeled peptides with C18 membranes, and then LC–MS/MS was applied for target protein analysis (Figure 6a).^33−35^

Statistical analysis revealed sphingomyelin phosphodiesterase 1 (SMPD1) as the most robustly stabilized protein in the presence of **hLR** (Figure 6b). And the mRNA expression of SMPD1 in THLE-2 cells was higher than that in HepG2 cells (Figure S33), suggesting a promising target protein for **hLR**. Then knockout (KO) of SMPD1 by CRISPR/Cas9 in the THLE-2 cells resulted in weaker fluorescence than the control THLE-2 cells shown in the fluorescent images, which confirmed that SMPD1 is the target protein of **hLR** (Figure 6c,d).

## Conclusion

Herein, we proposed a novel imaging modality by developing two different fluorescent probes, offering complementary imaging for the precise diagnosis of liver cancer. **cLG** was selective for liver cancer cells, whereas **hLR** was selective for healthy liver cells. These two probes selectively stained the targeted cells in the liver section, which was confirmed by H&E and antibody staining. In addition, the use of a cocktail of these two probes provided high contrast between the cancerous region and the normal region, enabling accurate cancer imaging. Finally, we identified the SLC27A2 transporter as the gating target of **cLG** through a systematic transporter screen using a CRISPR activation library. In addition, the binding target of **hLR** was verified as SMPD1 by thermal proteome profiling. Overall, the development of these two highly specific probes provides a unique diagnostic tool for cancer disease, even for fluorescence-guided surgery.

## Supporting Information Available

Experimental procedure of cell culture and biochemistry analysis, organic synthesis and characterization of compounds, optical properties, toxicity, selectivity, localization, *ex vivo* imaging of probes, SLC-CRISPRa screening of cLG (PDF)
