# Correction to “A Pair of Fluorescent Probes Enabling Precise Diagnosis of Liver Cancer by Complementary Imaging”

**DOI:** 10.1021/acscentsci.5c00250

**Published:** 2025-03-04

**Authors:** Min Gao, Sun Hyeok Lee, Haw-Young Kwon, Larissa Miasiro Ciaramicoli, Eunsol Jo, Young Hyun Yu, Fengming Li, Beomsue Kim, Kyungtae Hong, Jun-Seok Lee, Namhui Kim, Yoojin Oh, Chun Young Im, Chris Soon Heng Tan, Hyung-Ho Ha, Young-Tae Chang

We would like to add Grant No. RS-2024-00413760 to the Acknowledgments. The corrected version of the Acknowledgments is as given below.

We declare that the errata statement is true and valid, and this modification will not have any impact on the experimental conclusion. The other elements of the article remain the same, and the interpretation of the results remains unchanged.

