# Supplementary material for: A Pair of Fluorescent Probes Enabling Precise Diagnosis of Liver Cancer by Complementary Imaging
Source: ACS Cent Sci. 2024 Dec 16;11(1):76–83. doi: 10.1021/acscentsci.4c01822 (PMC11758269; doi:10.1021/acscentsci.4c01822)
Supplement: Supplementary file 1 — oc4c01822_si_001.pdf [file oc4c01822_si_001.pdf]

Supporting Information

A Pair of Fluorescent Probes Enabling Precise Diagnosis of Liver Cancer by Complementary Imaging

Min Gao,*[a] Sun Hyeok Lee,[b] Haw-Young Kwon,[c] Larissa Miasiro Ciaramicoli,[c] Eunsol Jo,[d] Young Hyun Yu,[d] Fengming Li,[e] Beomsue Kim,[f] Kyungtae Hong,[g] Jun-Seok Lee,[h] Namhui Kim,[i] Yoojin Oh,[i] Chun Young Im,[i] Chris Soon Heng Tan,*[e] Hyung-Ho Ha,*[d] Young-Tae Chang*[c]

[a] School of Chemistry and Chemical Engineering, Linyi University, Linyi 276005, P. R. China

[b] School of Interdisciplinary Bioscience and Bioengineering, Pohang University of Science and Technology (POSTECH), Pohang 37673, Republic of Korea

[c] Department of Chemistry, Pohang University of Science and Technology (POSTECH), Pohang 37673, Republic of Korea

[d] College of Pharmacy and Research Institute of Life and Pharmaceutical Sciences, Sunchon National University, Sunchon 57922, Republic of Korea

[e] Department of Chemistry, Southern University of Science and Technology, Shenzhen 518055, P. R. China

[f] Neural Circuits Research Group, Korea Brain Research Institute, Daegu 41062, Republic of Korea

[g] Bio-Med Program, KIST-School UST, Biomedical Research Division, Korea Institute of Science and Technology, Seoul 02792, Republic of Korea

[h] Department of Pharmacology, Korea University, Seoul 02841, Republic of Korea

[i] New Drug Development Center, Daegu-Gyeongbuk Medical Innovation Foundation(K-MEDIhub), Daegu 41061, Republic of Korea

E-mail: gaomin@lyu.edu.cn, christan@sustech.edu.cn, hhha@scnu.ac.kr, ytchang@postech.ac.kr

Table of contents:

1. Experimental procedures

2. Organic synthesis and characterization of compounds

3. Optical properties of of cLG

4. Optical properties of of hLR

5. Cytotoxicity of cLG and hLR in cells

6. Cell selectivity test

7. Cell localization

8. Mouse model of DEN-induced liver cancer

9. *Ex vivo* imaging of cLG/hLR.

10. Fluorescence enrichment of the cLG stained SLC-CRISPRa

11. mRNA expression of SMPD1

12. Reference

1. Experimental procedures

Cell culture for screening.

The THLE-2 and HepG2 cells were purchased from American Type Culture Collection (ATCC). THLE-2 cells were cultured in the media of BEGM (BEGM Bullet Kit; CC3170) in an atmosphere of 5 % CO_2 and 95 % air at 37 °C. HepG2 cells were cultured in the ATCC-formulated Eagle's Minimum Essential Medium (EMEM) supplemented with 10 % Fetal Bovine Serum (FBS) and 1% Penicillin Streptomycin (PS) in an atmosphere of 5 % CO_2 and 95 % air at 37 °C.

Image-based High Throughput Screening (HTS).

For the cell screening, THLE-2 and HepG2 cells were placed on 384-well plate and incubated with 1 μM of library compounds in duplicate at 37 °C. After 1 hour incubation, fluorescence image and bright-field images were acquired by the Operetta CLS (Perkin Elmer, Waltham, MA, USA) using a 20x objective. Data analysis was processed using Harmony 4.8 analysis software (Perkin Elmer, Waltham, MA, USA). Compounds with higher fluorescence staining in HepG2 cells over THLE-2 cells were selected as the primary candidates. After 3 repeated screenings, the candidate with the best contrast and highest reproducibility was chosen as the final probe for HepG2 cells. The criterion was opposite for hit compound of THLE-2 cell. The probe for THLE-2 cell showed higher fluorescence intensity in THLE-2 cells over HepG2 cells.

Flow cytometry analysis.

The cells were collected and treated under completed media containing **cLG** (1 μM) or **hLR** (1 μM) for 1 h at 37 °C. Flow cytometry was performed by the acquisition of 10,000 events. Flow cytometry was conducted on S3e cell sorter (Bio-Rad). Data analysis was performed using flowJo software.

Z-factor calculation.

The Z-factor is defined in terms of four parameters: the means (μ) and standard deviations (σ) of samples (s) and controls (c). Given these values (μ_s, σ_s, and μ_c, σ_c), the Z-factor is defined as:

$$\text{Z-factor} = 1 - \frac{3(\sigma_s + \sigma_c)}{|\mu_s - \mu_c|}$$

Liver cancer animal model.

All animal experimental protocols were performed in compliance with the Guidelines for the Pohang University of Science and Technology (POSTECH) Animal Care and Use committee (No. POSTECH-2020-0101). 12-15 days old male C57BL/6 mice were injected intraperitoneally with 25 mg/kg body weight of Diethylnitrosamine (DEN, Sigma-Aldrich, Munich, Germany).[1] 8.5-month-old male mice were served as controls. Mice were killed

8 months after DEN administration.

Tissue section imaging.

The livers were extracted from the normal and liver cancer model mice, and place in the OCT media at −20°C. Tissue sections were obtained using fluorescent microscopy (Axio Observer, ZEISS, Germany). The thickness of tissue section is 15 μm. Then the tissue section was ready for later staining and imaging. Prior to imaging, the tissue section was washed with PBS to remove OCT first.

Cytotoxicity of cLG and hLR.

CCK-8 assays were performed on THLE-2 and HepG2 cells to assess the cytotoxicity of various concentrations of cLG (0-200 μM) and hLR (0-200 μM). The cells were incubated with probe for 12 hours in the incubator. The absorbance was measured at 450 nm using a microplate reader.

CRISPR KO experiment.

The knockout experiment was performed in THLE-2 cells. SMPD1 knockout (KO) experiments were carried out by CRISPR/Cas9-editing with purified Cas9 protein (TrueCut Cas9 V2; ThermoFisher Scientific, Waltham, MA), Lipofectamine CRISPRMAX Transfection Regent (ThermoFisher Scientific, Waltham, MA), and sgRNAs of SMPD1 (Genscript, Nanjing, China, Target sequence: AGCGCGACAATGCCCCGCTA). The image was taken by ECLIPSE Ti2 Iv-storm (Nikon, Tokyo, Japan). The three separate experiments were proceeded with similar results. No data were excluded from the analyses.

RT-PCR.

RNA isolater Total RNA Extration Reagent (Vazyme, Nanjing, China) were used for total RNA extraction. cDNA from total RNA was generated using a PrimeScript™ RT Master Mix (Takara Biomedical Technology, Beijing, China). Then, real-time quantitative polymerase chain reaction (RT-qPCR) was performed using TB Green™ Premix Ex Taq™ II (Takara Biomedical Technology, Beijing, China) on Applied Biosystems 7300 Fast Real-Time PCR system (ThermoFisher Scientific, Waltham, MA) with the sequence of primers listed below. The relative mRNA levels of the genes were normalized with GAPDH. The primers for human SMPD1 were: F-5'-TCAAGGCTGAGAACGGGAAG-3' and R-5'- CGCCCCACTTGATTTTGGAG-3'. The primers for human GAPDH were: F-5'- TGGGGAAGATGTCATGTATGCTTTTACCCT-3' and R-5'-GATGCCCTCTTTCTCCAACCTGTTCTAGTG-3'.

Sample preparation for CESTA.

All experiments were performed based on the previous method.[2,3] THLE-2 cells were lysed in the lysis buffe r containing 50 mM HEPES (pH 7.5), 10 mM $MgCl_2$, 5 mM β-glycerophosphate, 0.1 mM activated Na_3VO_4, 1 m M TCEP, 0.2% (w/v) Lauryl maltose neopentyl glycol (LMNG) and EDTA-free protease inhibitor. The cell sus pension was subjected to five times flash-freezing in liquid nitrogen and rapid thawing in water to facilitate cell lysis. After centrifugation at 21000g for 20 min at 4 °C, the supernatant was transferred to a new tube and the pr otein concentration was measured with a BCA assay kit (Thermo Fisher Scientific, USA).

The cell lysate was first incubated with 20 μM hLR at room temperature for 5 min and then heated at 52 °C for 3 min. Three biological replicates were performed. And the cell lysate was treated with an equivalent amount of DMSO alone as a vehicle.

The SISPROT workflow was used to prepare collected samples for MS analysis. Proteins were denatured in a solution of 0.1% formic acid (FA) to acidify the sample. The SISPROT digestion device was filled with C18 disk (3 M Empore) and SCX/SAX mixed beads (1 mg, SCX: SAX = 1:1, Biosystems) into a standard 200 mL pipet tip. SISPROT tip was washed with methanol and 100 mM PCB potassium citrate buffer (pH 3) with 10 mM PCB to balance the tip. 5 μg of protein from 37 ℃ sample but obtained same volume (as the 37 ℃ sample) of drug-treated samples for processing with acidification (final concentration is 0.1% (v/v) formic acid) and then loaded each sample on a SISPROT tip. Each tip was washed with 20% (v/v) acetonitrile (ACN) in 8 mM potassium citrate buffer (pH 3) and ACN and incubate with 10 mM Tris(2-carboxyethyl) phosphine hydrochloride (TCEP) for 15 min at room temperature. The tips were then washed with 50 mM Tris-HCl, pH 8, and digestion solution (0.1 mg/mL trypsin (Promega) in 10 mM iodoacetamide, 50 mM Tris-HCl, pH 8) was added with incubation at 37℃ for 60 min. After digestion, peptides were washed with 500 mM NaCl from mixed beads to C18 before TMTpro labeling for 60 min. After labeling, 1%(v/v) FA was added and the peptides were washed with 80% (v/v) ACN, 0.5% (v/v) acetic acid for collection. All labeled peptide samples were mixed and lyophilized.

Peptide fractionation was performed after mixing of 8 samples labelled with TMTpro reagents. We used a 200 mL pipette tip filled with C18 membrane as a carrier for fractionation. The C18 device was activated with methanol and 80%(v/v) ACN, 0.5% (v/v) acetic acid, followed by 1% (v/v) FA to wash off the excess methanol solution. The lyophilized samples were diluted with 1% (v/v) FA and loaded onto a C18 apparatus. The C18 device was washed with 1% (v/v) FA solution and 5mMammonium formate, respectively. The collection bottles were then replaced with the collection devices with (3%, 5%, 7%, 9%, 11%, 13%, 15 %, 17%, 19%, 21%, 23%, 24%, 26%, 28%, 30%, 35%, 40%, 80%) ACN in 5mMammonium formate, pH 10. The effluent collected from 18 peptide fractions are (3%, 15%, 26%, and 50%), (5%, 17%, 28%, and 55%), (7%, 19%, 30%, and 60%), (9%, 21%, 35%, and 65%), (11%, 23%, 40%, and 70%), and (13%, 24%, 45%, 80%) ACN, which were then mixed together to get 6 fractions. Finally, the 6 fractions were lyophilized to dryness for MS analysis.

The peptides were analyzed by an Orbitrap Exploris 480 coupled with a U3000 HPLC system (Thermo Fisher Scientific) and a 20 cm × 100 μm i.d. analytical column in-house packed with C18 beads (1.9 μm/120 Å, Dr. Maisch GmbH). Samples were dissolved in buffer A [0.1% (v/v) FA in water]. A 135 min separation gradient was configured as 6−10% buffer B [100% (v/v) ACN in buffer B] in 2 min, 10−28% buffer B in 105 min, 28−40% buffer B in 11 min, and 40−97% buffer B in 2 min, followed by 7 min washing with 1% buffer B. MS parameters were as follows: Full MS scans over the *m/z* range of 350−1200 were performed at a resolution of 60,000. The Orbitrap Exploris 480 was operated in a data-dependent mode. Full scan and top 50 MS/MS scans were acquired per cycle with an MS1 scan resolution of 60,000 and an MS/MS scan resolution of 30,000 with the turbo-TMT option. HCD fragmentation was set with a normalized collision energy (NCE) of 38 and a dynamic exclusion of 45 s.

The acquired raw files were analyzed with Proteome Discoverer software (version 2.4.0) and searched against a database of human proteins, the Human FASTA database from UniProt (reviewed database entries, downloaded

on July 30, 2024). Precursor mass tolerance was set as 20 ppm, and fragment ions were searched at 20 mmu. Peptides were searched using the fully tryptic cleavage constraint, with up to two missed cleavages. Carbamidomethylation (C, + 57.02 Da) and TMTpro (N, K, + 304.207 Da) were used as fixed modifications. FDR control is set to 1% at the PSM and peptide levels. The coisolation threshold for report ion quantification is set to 50%.

Statistical Analysis.

The data are presented as mean ± standard deviation (SD). The experiments have been repeated minimally three times. The error bars are derived from SD.

2. Materials and methods for cLG and hLR

All used compounds and solvents were purchased from Alfa Aesar (Haverhill, MA, USA), Acros Organics (Fair Lawn, NJ, USA), Combi-Blocks (San Diego, CA, USA), Cambridge Isotope Laboratories (Tewksbury, MA, USA), Sigma Aldrich (St. Louis, MO, USA), and Samchun chemicals (Seoul, Republic of Korea). 2-Chlorotrityl alcohol resin (1.37 mmol/g) was purchased from BeadTech Inc. (Ansan, Republic of Korea). TLC Silica gel 60 F254 (Merck) was used for analytical TLC and silica gel 60 (0.040-0.063 mm) (Merck) was used for silica gel chromatography. The optical properties were measured on microplate reader (Molecular devices, SpectraMax M2e). The relative fluorescence quantum yield method was chosen, and fluorescein was exploited as the quantum yield standard.[4] The quantum yield was calculated according to the equation (1). For **cLG** synthesis, ^{1}H and ^{13}C NMR spectra were obtained from Brucker AVANCE III HD 500 and AVANCE III HD 850 and mass spectra were recorded on ion trap mass spectrometer (Thermo Scientific, LTQ XL™). For CROS library, ^{1}H spectra were obtained from JEOL 600 MHz NMR spectrometer and LC-MS related data was obtained from HPLC (Agilent, 1260 series) with DAD (diode array detector) and single quadrupole mass (Agilent, 6100 series). The HR-MS measurement (JEOL JMS700) was entrusted to Daegu center in Korea Basic Science Institute (KBSI).

$$\Phi_{fl}=\Phi_{ref}(m/m_{ref})(n/n_{ref})^2 \qquad (1)$$

Where the ref means the reference fluorophore. Φ is the fluorescence quantum yield, m is the slope of the trend line in the scatter plot (y axis, area under curve of fluorescence, x axis, absorbance), and n is the refractive index.

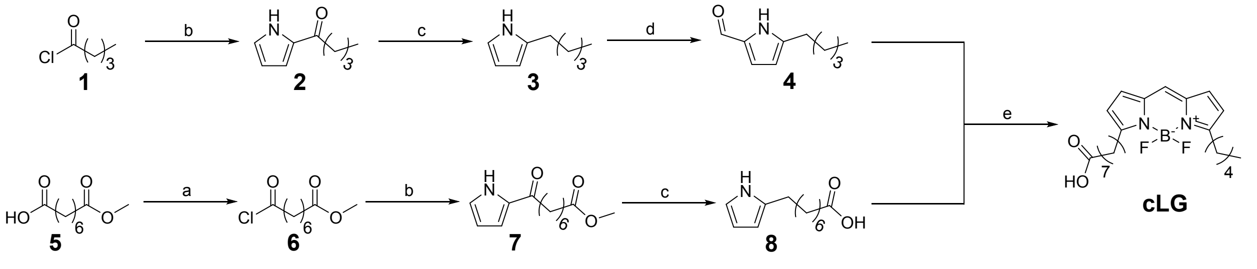

Scheme S1. Synthesis process for **cLG.** (a) $(COCl)_2$, DCM, 0 ℃→r.t., 2 h; (b) pyrrole, $AlCl_3$, DCM, r.t., overnight; (c) $NaBH_4$, IPA, reflux, 10 h; (d) i) $POCl_3$, DMF, DCE, 0 ℃, 15 min, ii) 80 ℃, 30 min iii) NaOAc in H_2O, rt→80 ℃, 20 min; (e) i) $POCl_3$, 0 ℃, 2 h ii) DMAN, BF_3•OEt_2, rt, 3 h.

Synthesis procedure for cLG compound

For **cLG** synthesis, the detail procedure was followed with the previous study.[5]

Synthesis of 1-(1*H*-pyrrol-2-yl)pentan-1-one (2).

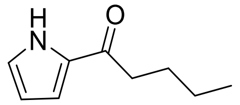

The procedure followed General procedure I in the precedent[5], and **1** (1.60 ml, 13.4 mmol), pyrrole (1.03 ml, 14.8 mmol), $AlCl_3$ (2.14 g, 16.08 mmol) and DCM (26.8 ml) were used for Friedel-Craft acylation. The crude mixture was purified via silica gel chromatography using a gradient of EA:hexane from 1:10 to 1:3 to obtain pinkish crystal (1.3 g, 64.1%). ^1H NMR (500 MHz, $CDCl_3$): δ (ppm) 10.94 (br, 1H), 7.07 (br, 1H), 6.95 (br, 1H), 6.26 (br, 1H), 2.82 (t, 2H, J = 7.45 Hz), 1.77 (qu, 2H, J = 7.35 Hz), 1.45 (sext, 2H, J = 7.15 Hz), 0.97 (t, 3H, J = 7.4 Hz). ^{13}C NMR (125 MHz, $CDCl_3$): δ (ppm) 191.61, 131.94, 125.39, 116.75, 110.22, 37.67, 27.54, 22.50, 13.83.

Synthesis of 5-pentyl-1*H*-pyrrole-2-carbaldehyde (4).

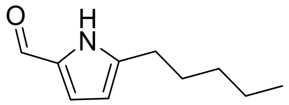

The reduction followed General procedure II in the precedent[5]. **2** (1.3 g, 8.6 mmol), $NaBH_4$ (2.28 g, 60.2 mmol) and IPA (18.8 ml) were used for the reduction and the residue was utilized for the next step without additional purification. The Vilsmeier reaction followed General procedure III in the precedent[3]. **3** (*in situ*), DMF (0.73 ml, 9.46 mmol), $POCl_3$ (0.88 ml, 9.46 mmol), DCE (34 ml), NaOAc (3.17 g, 38.7 mmol), and H_2O (20 ml) were used for the aldehyde introduction and the residue was purified via silica gel chromatography using EA:Hexane=1:8 to obtain brown oil (657 mg, 46.2%). ^1H NMR (500 MHz, $CDCl_3$): δ (ppm) 10.74 (br, 1H), 9.34 (s, 1H), 6.92 (br, 1H), 6.08 (br, 1H), 2.71 (t, 2H, J = 7.7 Hz), 1.68 (qu, 2H, J = 6.8 Hz), 1.32 (br, 4H), 0.89 (t, 3H, J = 6.8 Hz). ^{13}C NMR (125 MHz, $CDCl_3$): δ (ppm) 178.18, 144.61, 131.88, 123.60, 109.58, 31.46, 28.84, 27.86, 22.45, 14.03.

Synthesis of methyl 8-oxo-8-(1*H*-pyrrol-2-yl)octanoate (7).

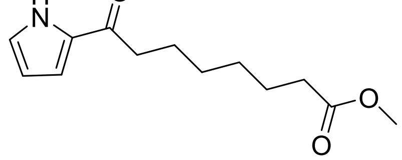

The procedure followed General procedure I in the precedent[5], and **5** (5.06 g, 26.9 mmol), $(COCl)_2$ (2.8 ml, 32.3 mmol), DMF (0.2 ml, 2.69 mmol) and DCM (26 ml) were used to prepare acid chloride. **6** (*in situ*), pyrrole (2.06 ml, 29.7 mmol), $AlCl_3$ (4.3 g, 32.3 mmol) and DCM (38 ml) were used for Friedel-Craft acylation. The

crude mixture was purified via silica gel chromatography using a gradient of EA:hexane from 1:10 to 1:3 to obtain white solid (1.88 g, 29.5%). ^{1}H NMR (500 MHz, $CDCl_3$): δ (ppm) 10.33 (br, 1H), 7.04-7.05 (m, 1H), 6.91-6.93 (m, 1H), 6.25-6.26 (m, 1H), 3.66 (s, 3H), 2.78 (t, 2H, J = 7.4 Hz), 2.32 (t, 2H, J = 7.45 Hz), 1.76 (qu, 2H, J = 7.45 Hz), 1.66 (qu, 2H, J = 7.55 Hz), 1.32-1.41 (m, 4H). ^{13}C NMR (125 MHz, $CDCl_3$): δ (ppm) 191.19, 174.24, 132.00, 125.02, 116.44, 110.41, 51.48, 37.86, 34.00, 29.04, 28.93, 25.11, 24.77. ESI-MS $[M+H]^+$: m/z calcd for $C_{13}H_{20}NO_3$ 238.14, found 238.33.

Synthesis of 8-(1*H*-pyrrol-2-yl)octanoic acid (8).

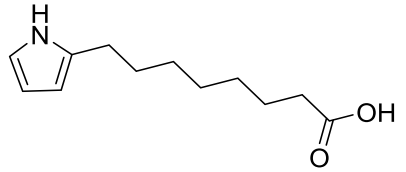

The reduction followed General procedure II in the precedent[5]. **7** (1.72 g, 8.79 mmol), $NaBH_4$ (2.33 g, 61.6 mmol) and IPA (17 ml) were used for the reduction and the residue was purified via silica gel chromatography using a gradient of EA:hexane from 1:10 to 1:1 to obtain brown solid (1.04 g, 70.7%). ^{1}H NMR (500 MHz, $CDCl_3$): δ (ppm) 7.90 (br, 1H), 6.64-6.5 (m, 1H), 6.11-6.12 (m, 1H), 5.90 (br, 1H), 2.59 (t, 2H, J = 7.6 Hz), 2.35 (t, 2H, J = 7.45 Hz), 1.64 (sext, 4H, J = 7.25 Hz), 1.33 (br, 6H). ^{13}C NMR (214 MHz, $CDCl_3$): δ (ppm) 180.22, 132.82, 116.12, 108.36, 105.01, 34.13, 29.67, 29.20, 29.10, 29.03, 27.79, 24.72. ESI-MS $[M-H]^-$: m/z calcd for $C_{12}H_{18}NO_2$ 208.13, found 208.17.

Synthesis of cLG

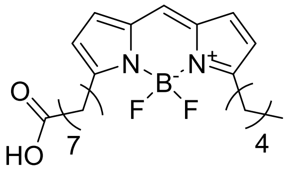

The reaction followed General procedure IV in the precedent[5]. **4** (24 mg, 0.15 mmole), **8** (21 mg, 0.15 mmole), $POCl_3$ (14 µl, 0.15 mmole) and DCM:hexane=1:1 (v/v) (3 ml) were used for the first step. DMAN (128 mg, 0.6 mmol) and BF_3•OEt_2 (0.1 ml, 0.84 mmol) were used for the second step. The residue was purified via silica gel chromatography using EA:Hexane=1:10 to EA:Hexane=1:3 with 0.1% AcOH to obtain orange red solid (7 mg, 15.6%). ^{1}H NMR (850 MHz, $CDCl_3$): δ (ppm) 7.05 (s, 1H), 6.95 (t, 2H, J = 3.74 Hz), 6.33 (dd, 2H, J = 4.17, 10.37 Hz), 2.99 (dt, 4H, J = 2.46, 8.84 Hz), 2.36 (t, 2H, J = 7.56 Hz), 1.71-1.75 (m, 4H), 1.66 (qu, 2H, J = 7.48 Hz), 1.35-1.44 (m, 10H), 0.91 (t, 3H, J = 7.31 Hz). ^{13}C NMR (214 MHz, $CDCl_3$): δ (ppm) 178.48, 163.39, 163.00, 134.52, 134.46, 130.10, 130.01, 127.03, 118.14, 118.04, 33.84, 31.86, 29.45, 29.13, 29.05, 28.94, 28.90, 28.57, 28.32, 24.79, 22.62, 14.11. LC-MS (ESI) $[M-H-2F]^+$: m/z calcd for $C_{22}H_{30}BN_2O_2$ 365.24, found 365.50; $[M-F]^+$: m/z calcd for $C_{22}H_{31}BFN_2O_2$ 385.25, found 385.42.

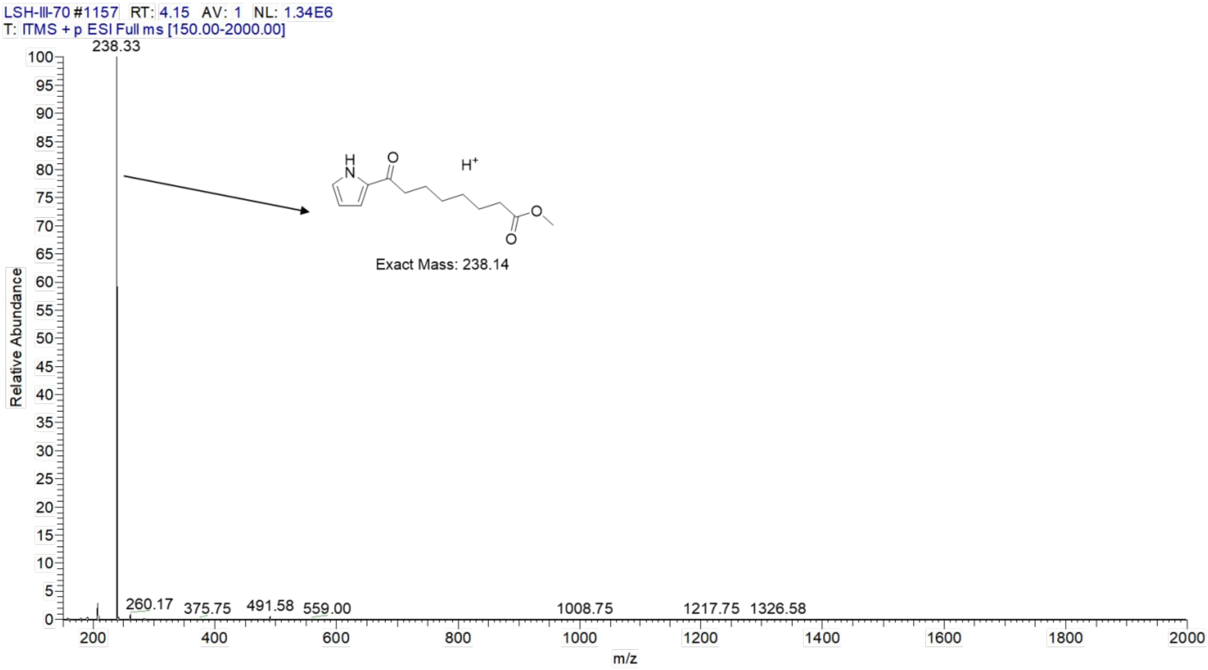

Figure S1. Mass spectrum of compound **7**.

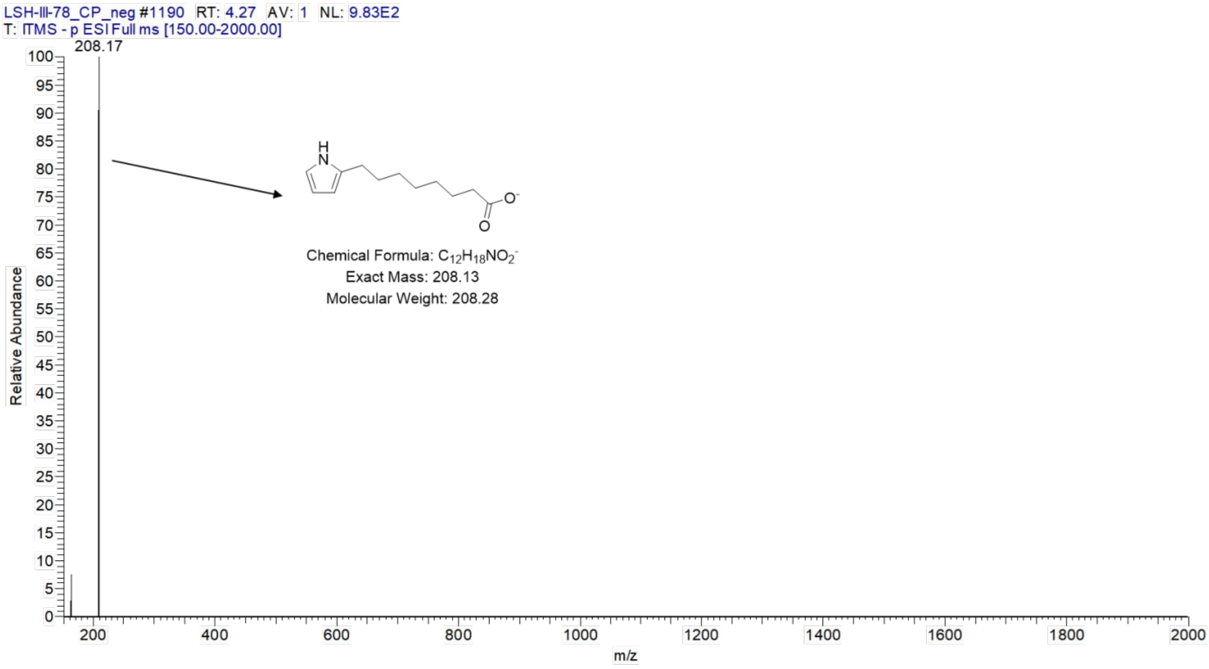

Figure S2. Mass spectrum of compound **8**.

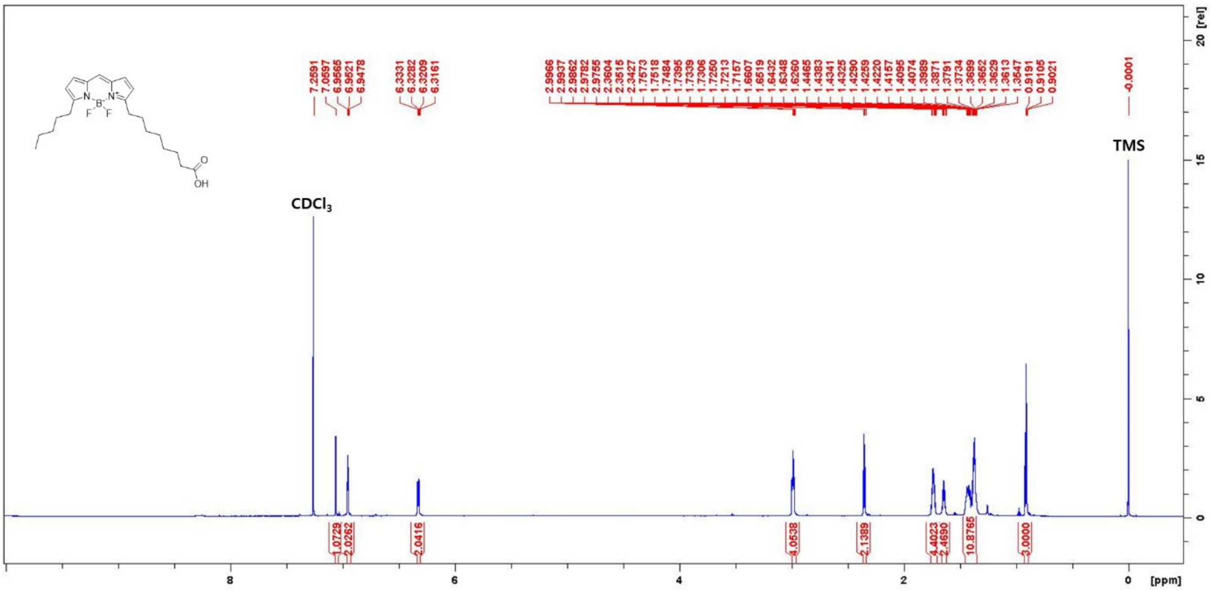

Figure S3. ^{1}H NMR of **cLG** (850 MHz, $CDCl_3$).

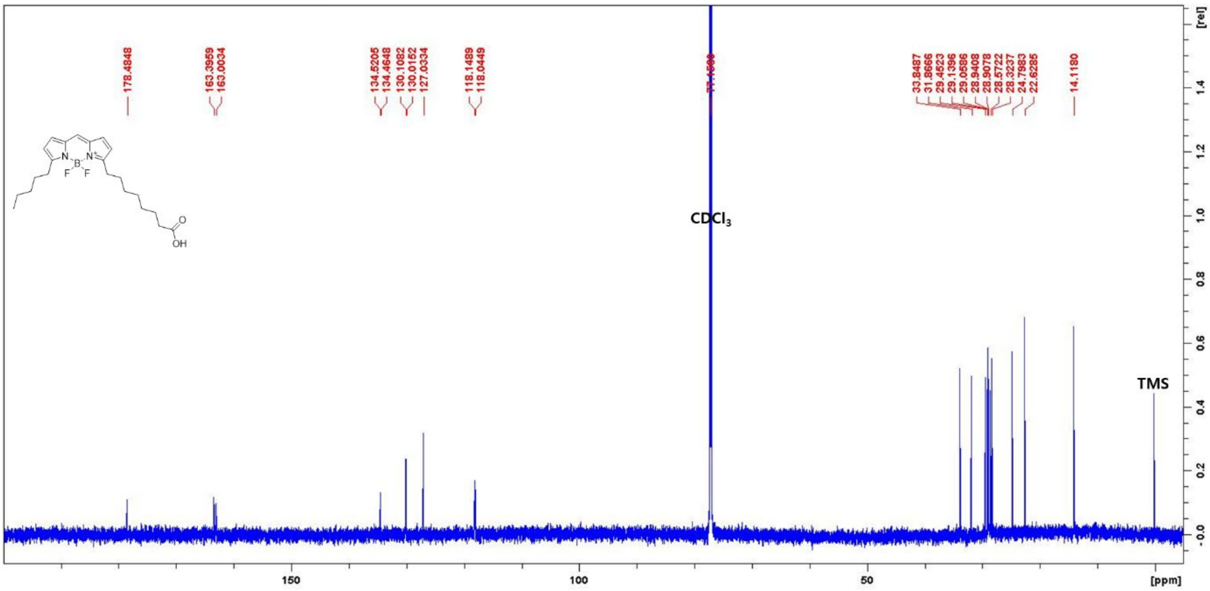

Figure S4. ^{13}C NMR of **cLG** (214 MHz, $CDCl_3$).

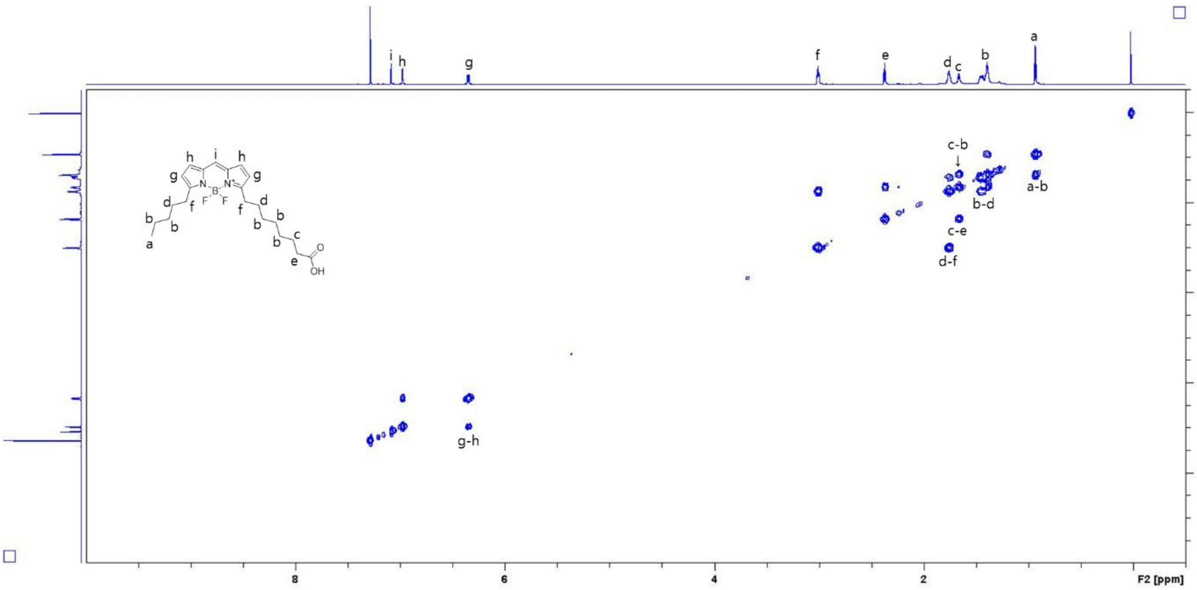

Figure S5. ^{1}H-^{1}H COSY of **cLG** (850 MHz, $CDCl_3$).

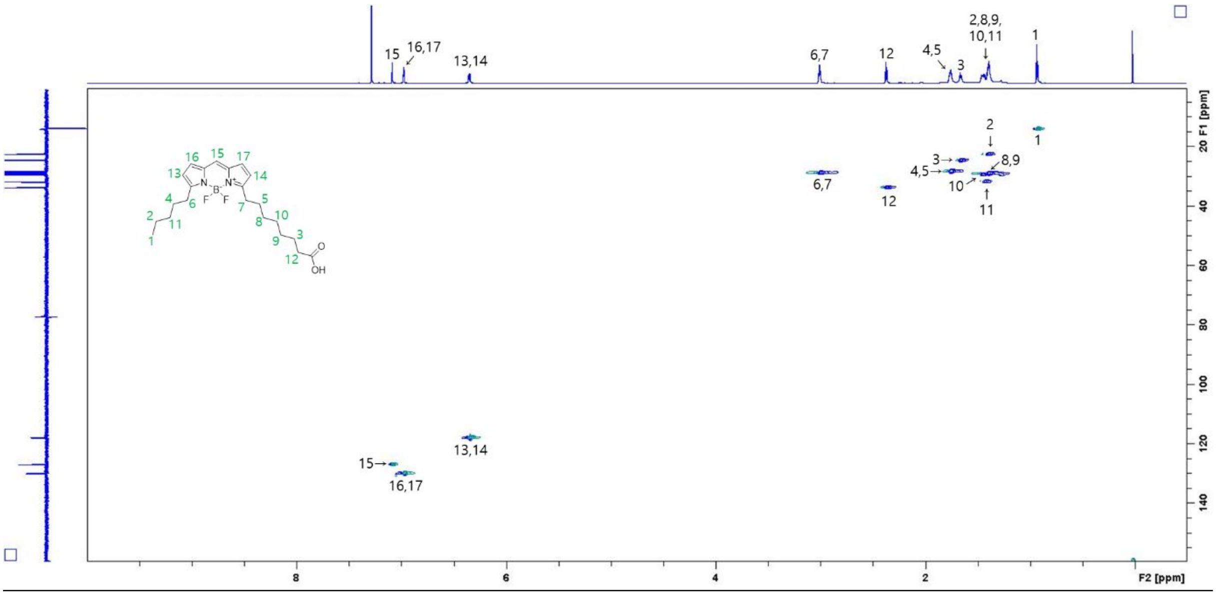

Figure S6. ^{1}H-^{13}C HSQC of **cLG** (850 MHz for ^{1}H and 214 MHz for ^{13}C, $CDCl_3$).

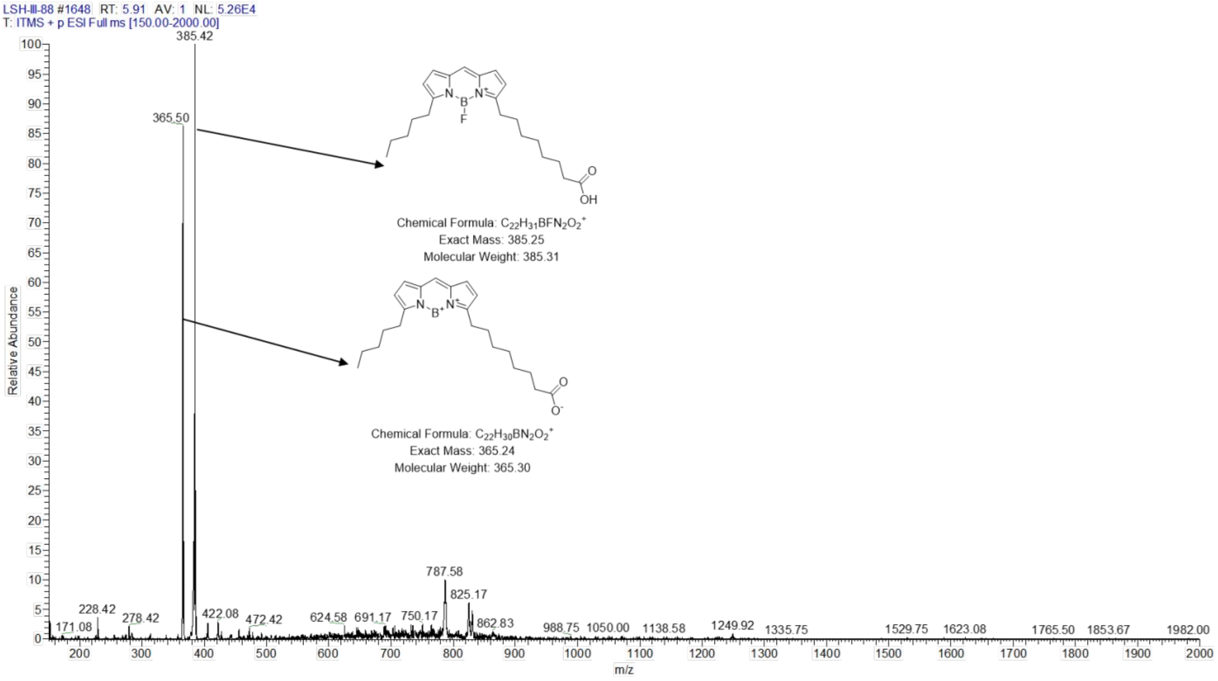

Figure S7. Mass spectrum of **cLG**.

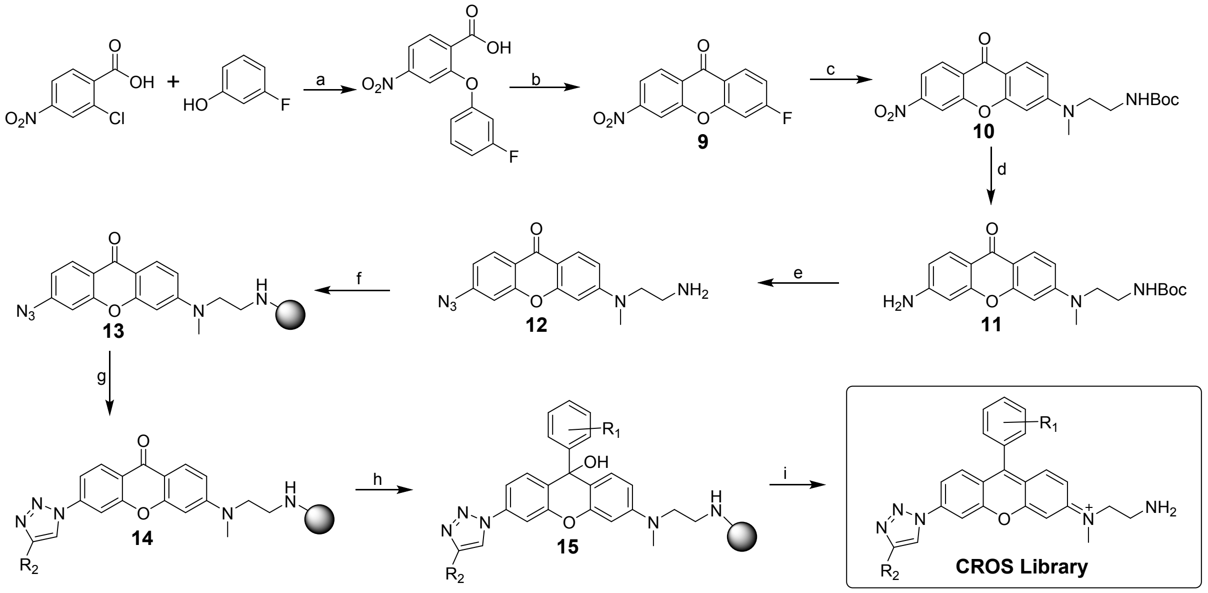

Scheme S2. Synthesis process for CROS library. (a) K_2CO_3, Cu, DMF, 140 °C, 8h; (b) H_2SO_4, 80 °C, 1h; (c) tert-butyl 2-(methylamino)ethylcarbamate, DMSO, 90 °C, 8h; (d) $N_2H_4 \cdot H_2O$, Pd/C, EtOH, 95 °C, 3h; (e) $NaNO_2$, AcOH, NaN_3, rt, 2h and then 10% TFA/DCM, rt, 2h; (f) Chlorotrityl chloride Resin, DMF, DIPEA, rt, 12h; (g) CuI, Ascorbic acid, Acetylene derivatives, DMF: Piperidine (8:2), rt, 8h; (h) Grignard Reagent, THF, 70 °C, 24h; (i) 2% TFA/DCM

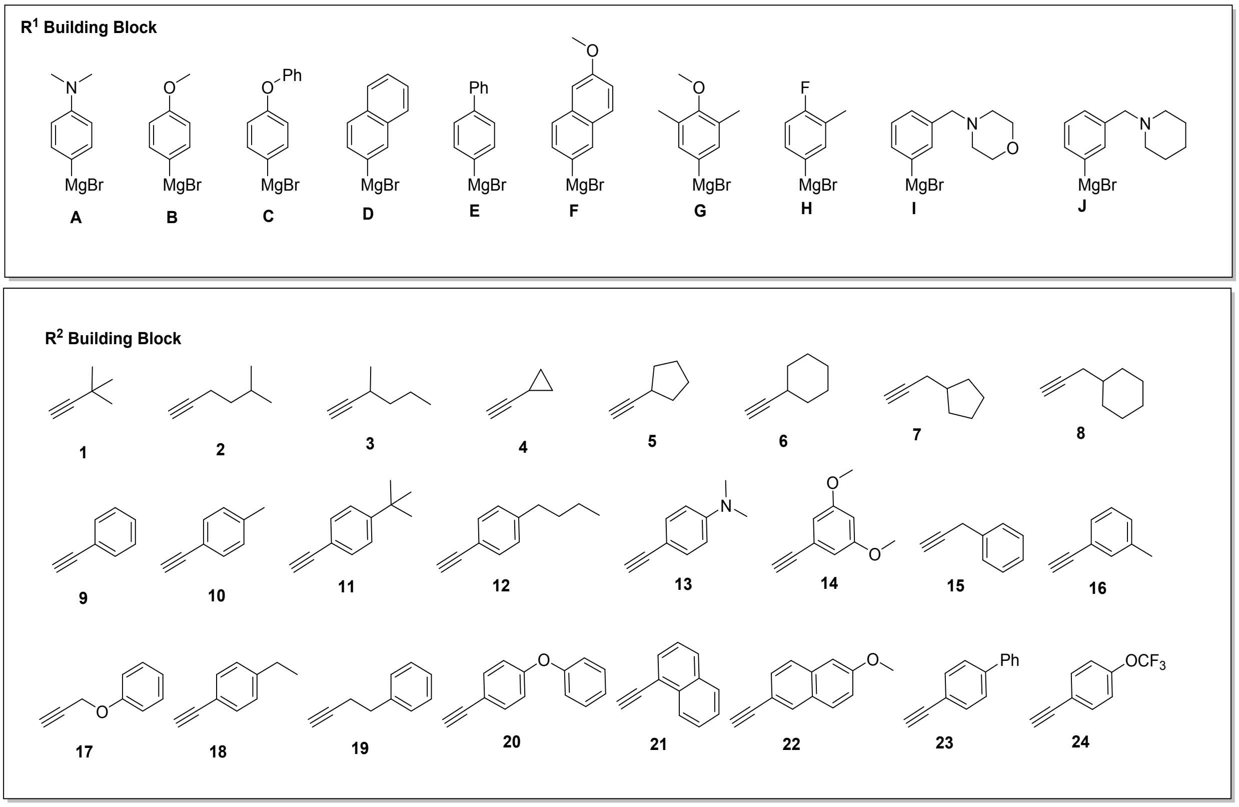

Chart S1. Building blocks for CROS library.

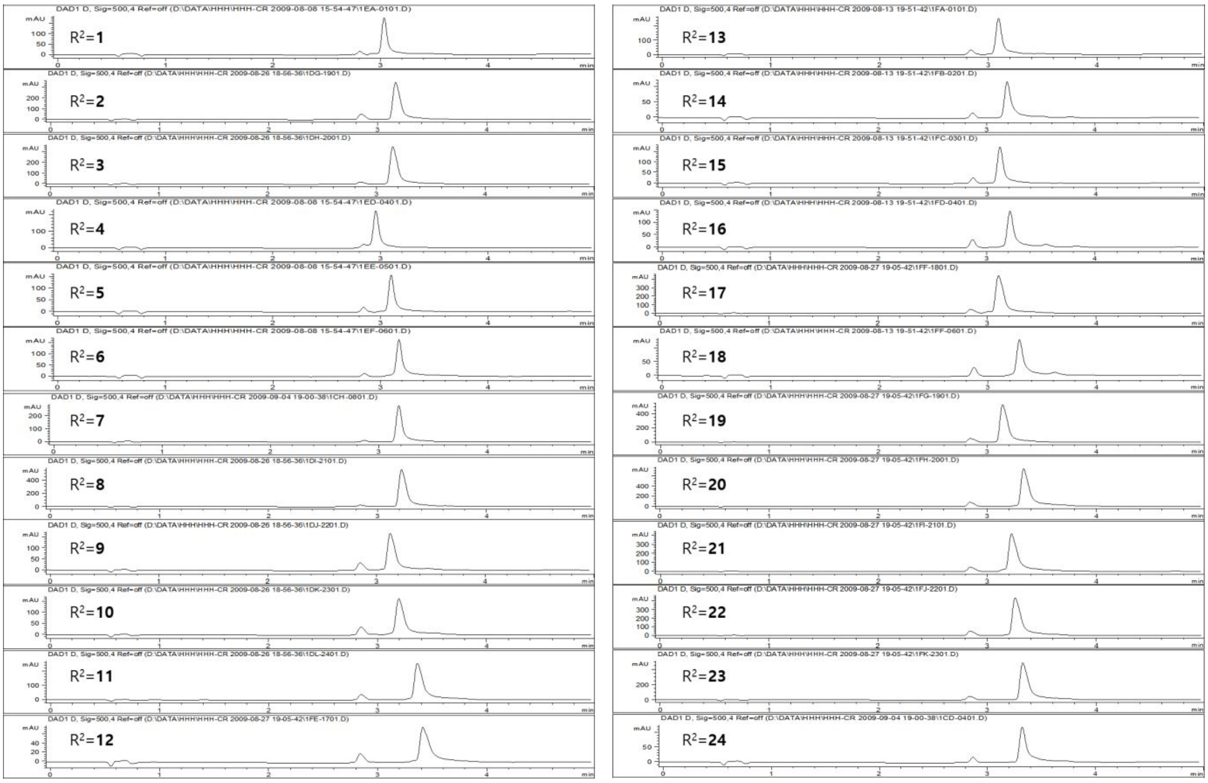

Figure S8. HPLC chromatography of CROS library compounds (**CROS-E1** to **CROS-E24**). All compounds were measured immediately following their detachment from the resin, without further purification. Left top to bottom: **CROS-E1** to **CROS-E12**. Right top to bottom: **CROS-E13** to **CROS-E24**. Absorbance wavelength: 500 nm. A: H_2O w 0.1% FA, B: ACN w 0.1% FA, gradient from 30%B to 100%B in 5 min; column: Phenomenex C18 Luna column (4.6 x 50 mm, 5 µm particle size), flow rate: 0.8 ml/min. The HPLC chromatographs were recorded on HPLC (Agilent 1200).

Synthetic Procedure for hLR

Synthesis of 3-fluoro-6-nitro-9H-xanthen-9-one (9)

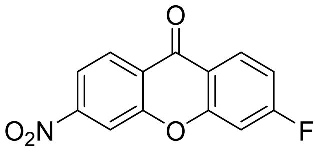

To a solution of 2-chloro-4-nitrobenzoic acid (3.0 g, 14.88 mmol) in DMF (40 ml) was added 3-fluorophenol (2.47 g, 16.38 mmol), potassium carbonate (3.08 g, 16.38 mmol) and copper Powder (102 mg, 1.61 mmol). After heating at 140 °C overnight the reaction mixture was then filtered through celite and washed with DMF. Once evaporating the DMF, 1 N HCl at 0 °C was added to it. The solution was stirred until the brown solid was formed. The solid was filtered off and washed with cold water to yield a brown solid (3.11 g, 75%). A crude solid was dissolved in concentrated sulfuric acid (20 ml) and heated at 80 °C for 1 hour. After cooling to room temperature, the reaction mixture was poured into ice (350 ml volume) and stirred for one hour. Then filter it and dry it in the fume hood (2.12 g, 73%). ^{1}H NMR (600 MHz, $CDCl_3$): δ (ppm) 8.51 (d, J = 8.6 Hz, 1H), 8.40-8.37 (m, 2H), 8.21 (dd, J = 2.2, 8.8 Hz, 1H), 7.25 (m, 1H), 7.21-7.18 (m, 1H). HR-MS (ESI) $[M+H]^+$: m/z calcd for $C_{13}H_7FNO_4$ 260.0354, found 260.0357 (Δm = 1.154 ppm).

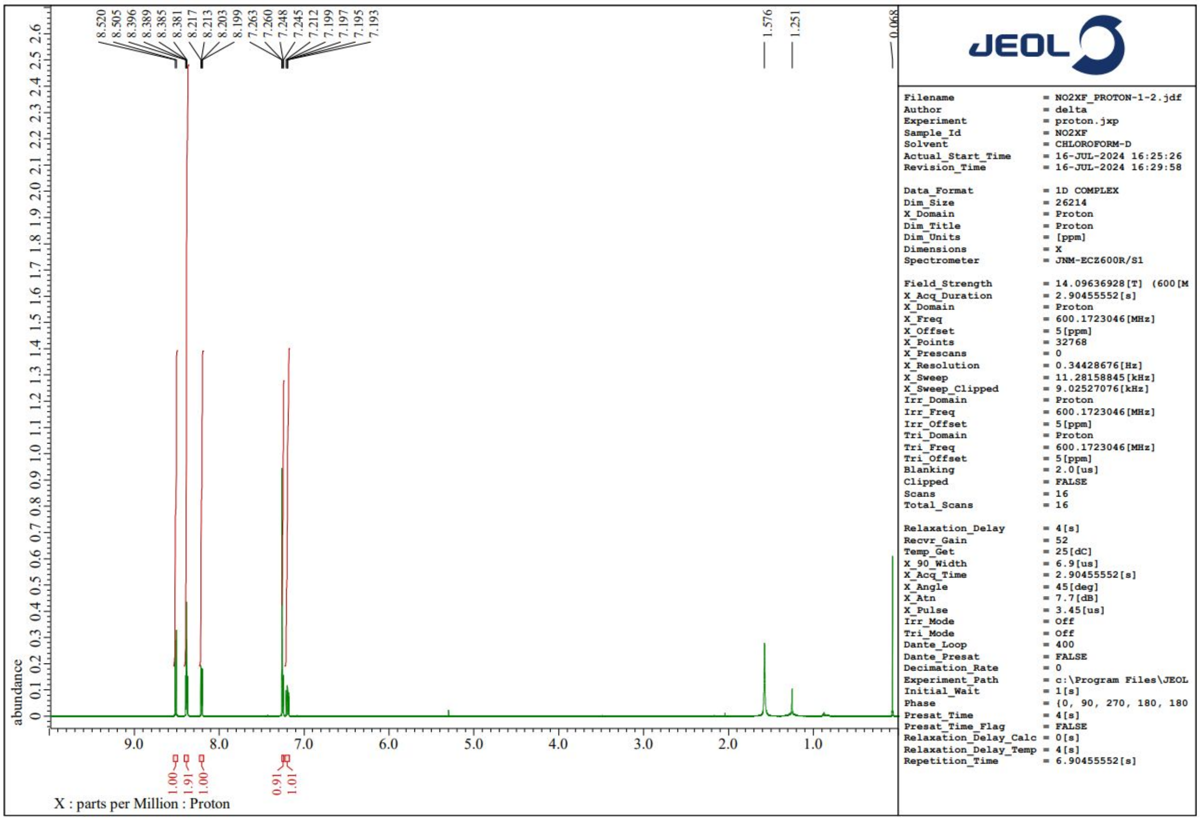

Figure S9. ^{1}H NMR of compound **9** (600 MHz, $CDCl_3$).

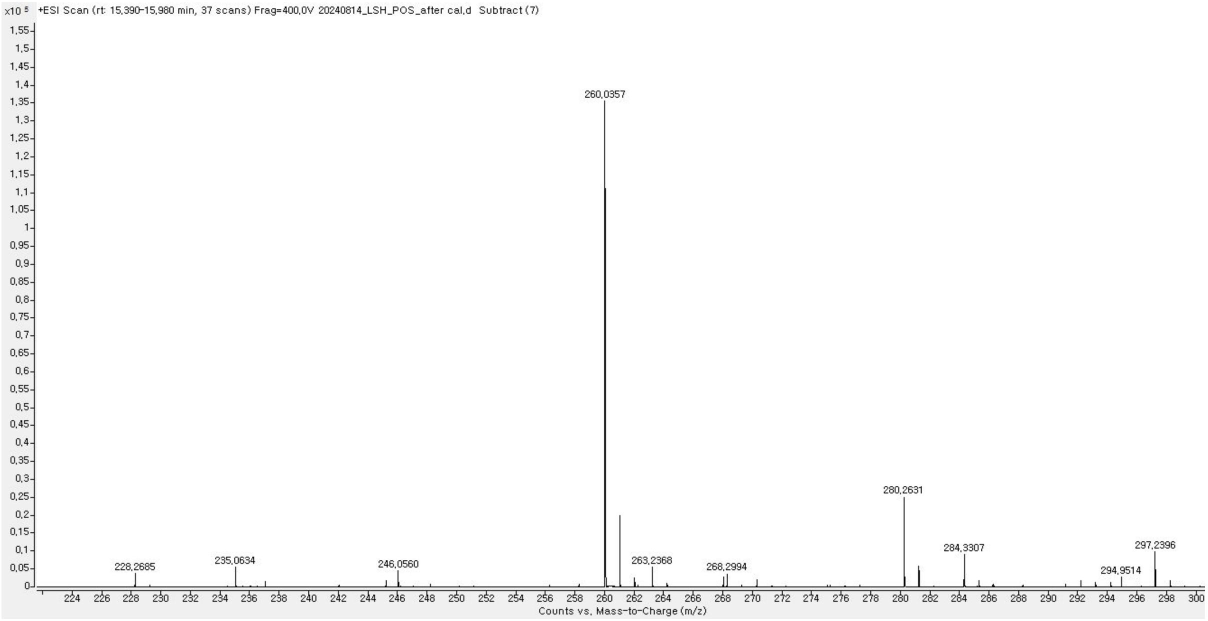

Figure S10. High resolution mass spectrum of compound **9**. The high resolution mass spectrum was recorded on quadrupole time of flight MS (Agilent 6560).

Synthesis of tert-butyl (2-(methyl(6-nitro-9-oxo-9H-xanthen-3-yl)amino)ethyl)carbamate (10)

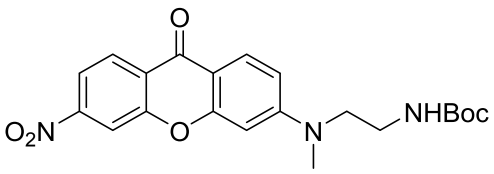

Compound **9** (2.12g, 8.17 mmol) was dissolved in DMSO (0.2 M) and the tert-butyl 2-(methylamino)ethylcarbamate amine (2.85g, 16.34 mmol) was added in one portion. The reaction mixture was heated to 90°C and stirred for 8 h. The solution was then cooled to r.t. and water was added. The precipitate was collected and washed with diethyl ether and water to render compound **10** (1.15g, 34%). ^{1}H NMR (600 MHz, $CDCl_3$): δ (ppm) 8.45 (d, J = 9.0 Hz, 1H), 8.26 (d, J = 2.1 Hz, 1H), 8.15-8.11 (m, 2H), 6.83 (dd, J = 2.2, 9.1 Hz, 1H), 6.59 (d, J = 2.1 Hz, 1H), 4.74 (s, NH), 3.65 (t, J = 6.0 Hz, 2H), 3.38 (q, J = 6.0 Hz, 2H), 3.15 (s, 3H), 1.43 (s, 9H). LC-MS (ESI) $[M+H]^+$: *m/z* calcd for $C_{21}H_{24}N_3O_6$ 414.2, found 414.2; $[2M+Na]^+$: *m/z* calcd for $C_{42}H_{46}N_6NaO_{12}$ 849.3, found 849.4.

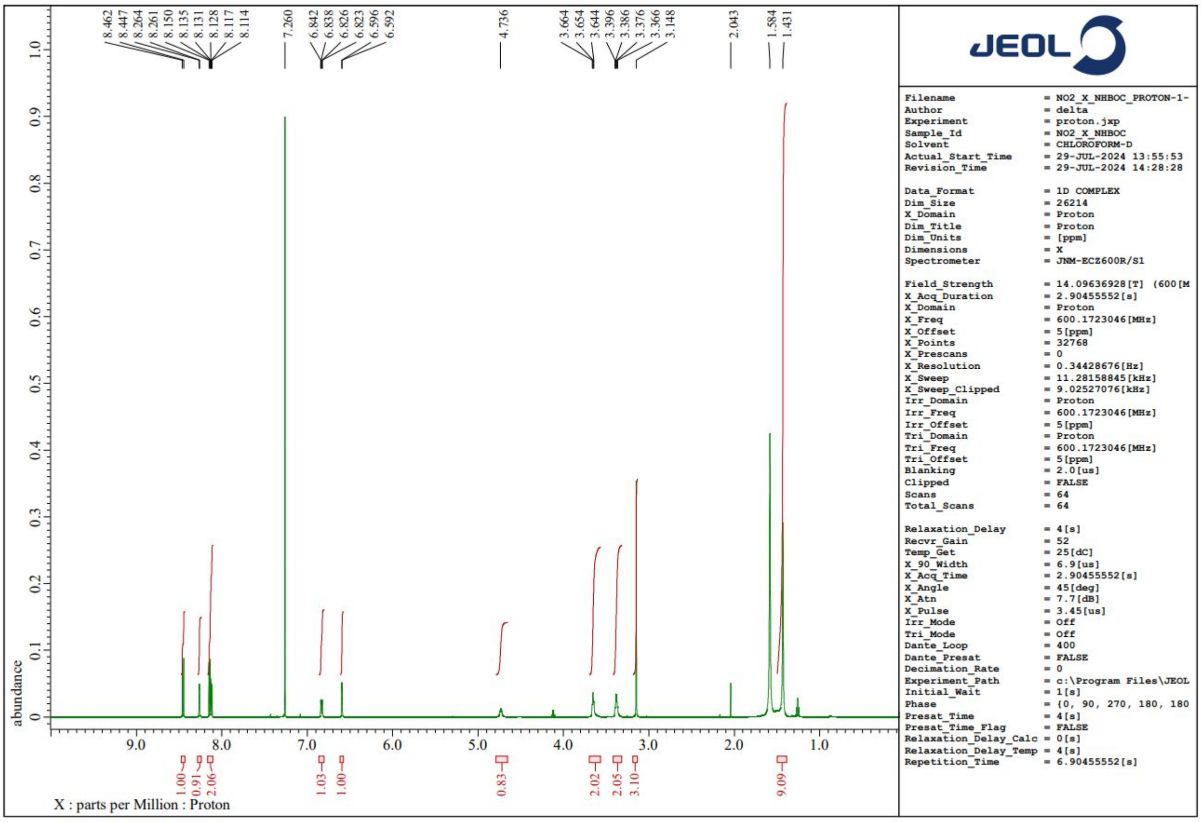

Figure S11. ^{1}H NMR of compound **10** (600 MHz, $CDCl_3$).

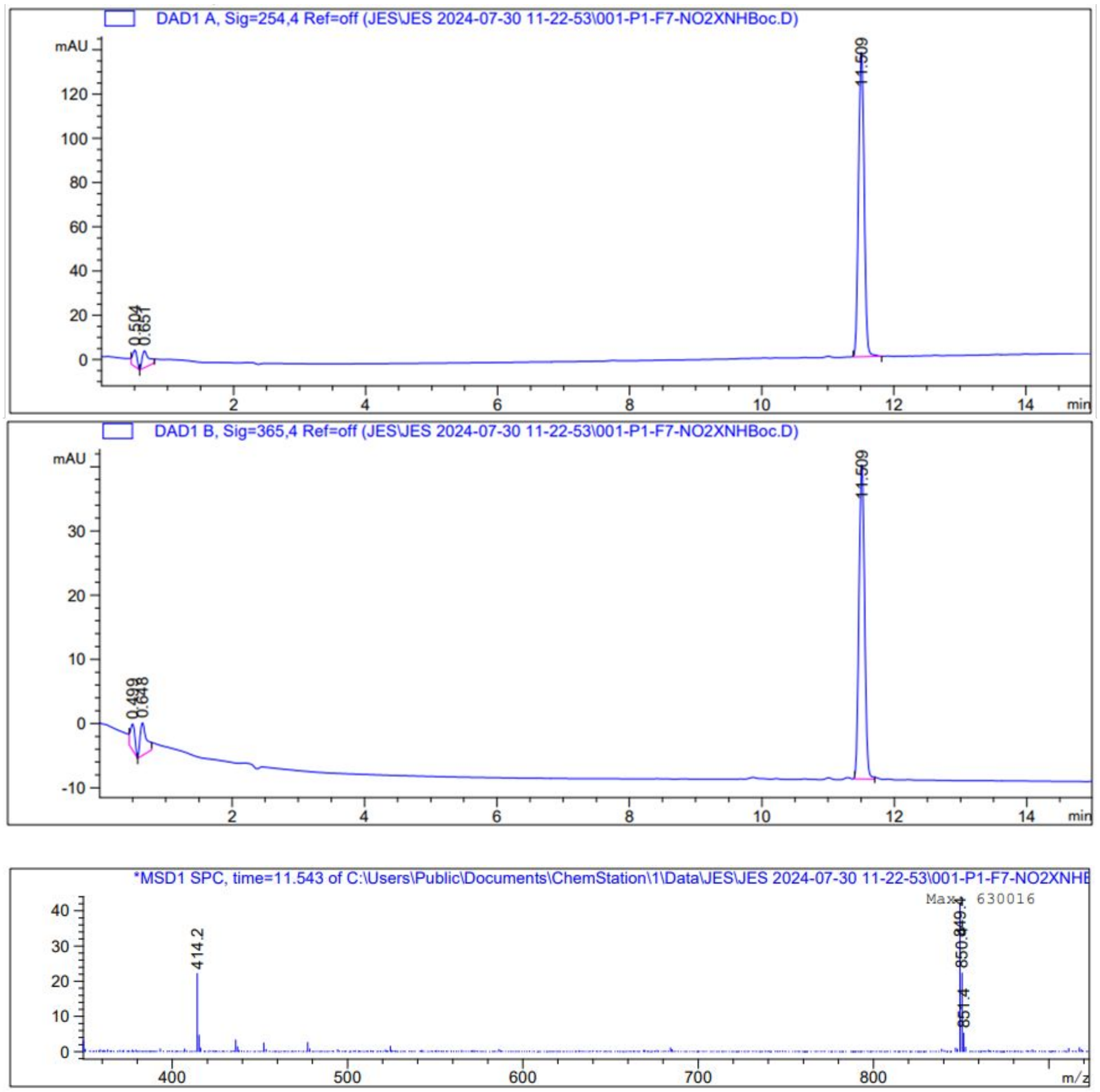

Figure S12. HPLC chromatography and mass spectrum of compound **10**. Top: HPLC chromatography of compound **10**. Absorbance wavelength: 254 and 365 nm (top to bottom). A: H_2O w 0.1% TFA, B: ACN w 0.1% TFA, gradient from 10%B to 99%B in 15 min; column: Agilent InfinityLab Poroshell 120 CS-C18 (2.1 x 50 mm, 2.7 µm particle size). Bottom: mass spectrum of compound **10** extracted from t_R = 11.54 min. Where t_R means retention time, and TFA is the abbreviation of trifluoroacetic acid.

Synthesis of tert-butyl (2-((6-azido-9-oxo-9H-xanthen-3-yl)(methyl)amino)ethyl)carbamate (12)

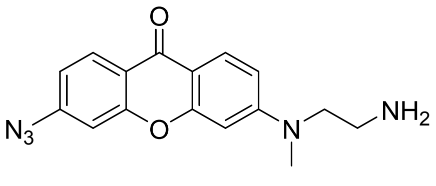

To a solution of **10** (1 g, 2.42 mmol) was added $N_2H_4 \cdot H_2O$ (1.21 g, 24.2 mmol) and Pd/C (200 mg, 20% of the compound) and heated at 95 °C for 3 hours. The hot solution was filtered through celite, and the solvents were removed under reduced pressure to obtain compound **11** (780 mg, 84%). The compound was used in the next step without further purification.

To a solution of **11** (780 mg, 2.03 mmol) in AcOH/H_2O (1:1), $NaNO_2$ (168 mg, 2.44 mmol) was added at 0 °C and stir it for 1 hour. The diazonium salt obtained was filtered and to the filtrate at 0 °C. NaN_3 (198 mg, 3.05 mmol) was added and stir it for 1.5 hour. The solution was then neutralized with saturated $NaHCO_3$ and extracted with DCM. After evaporating the solvent, the residue was subjected to flash column chromatography (30 to 40% EtOAc in Hexane) to obtain Boc-protected **12** (662 mg, 80%). 670 mg Boc-protected **12** (670 mg, 1.64 mmol) was added to 25 ml of 10% TFA in DCM and stirred at room temperature for one hour. The solution was then evaporated several times with DCM to obtain compound **12** (650 mg, 1.54 mmol, 94%) as TFA salt. The compound **12** was used directly in the loading reaction with 2-chloro-trityl chloride resin without further purification.

Loading 3-((2-aminoethyl)(methyl)amino)-6-azido-9H-xanthen-9-one to solid resin (13)

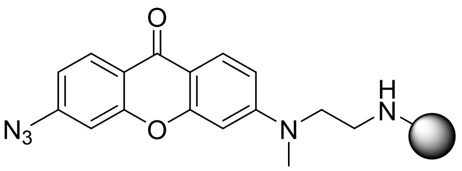

1 eq. of compound **12** was dissolved in 10 ml DMF/DCM (9:1) and DIEA was added to it. The solution was then added to 2-chlorotrityl chloride resin (0.6 mmol) suspended in dichloromethane (2 ml). After stirring for 12 h, the resin was filtered through 10 ml cartridge and washed with DMF (X5), MeOH (X10), and DCM (X10). The resin was then shaken with 20% MeOH in DMF for 2 hours. The resin again washed with DMF (X5), methanol (X5), and dichloromethane (X5) and dried using high vacuum for 2 hours. The loading was 80%.

General Procedure for click chemistry on Solid Support (14)

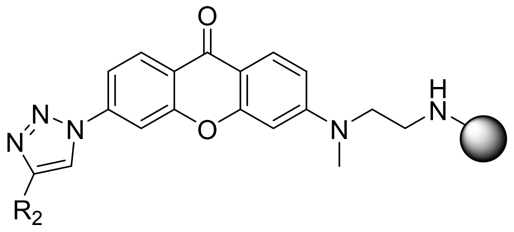

For each reaction, a resin (60 mg, 0.048 mmol) was suspended in 2 ml of DMF/Piperidine (4:1) in a 10 ml of syringe. 5 eq. of CuI (0.24 mmol) and 5 eq. of ascorbic acid (0.24 mmol) was then dissolved in 2 ml of the same

solvent and added to the resin along with the 5 eq. of alkynes (0.24 mmol). The reaction mixture was shaken for overnight at room temperature and the resin was filtered through 10ml cartridge and washed with DMF, 1% Sodium diethylthiocarbamate in DMF, 1% DIEA in DMF, 10% H_2O in DMF. Finally, the resin was washed with DMF (X5), MeOH (X5), and DCM (X5). LC-MS of compound **14** (R^2 building block 24) (ESI) $[M+H]^+$: *m/z* calcd for $C_{25}H_{21}F_3N_5O_3$ 496.2, found 496.4.

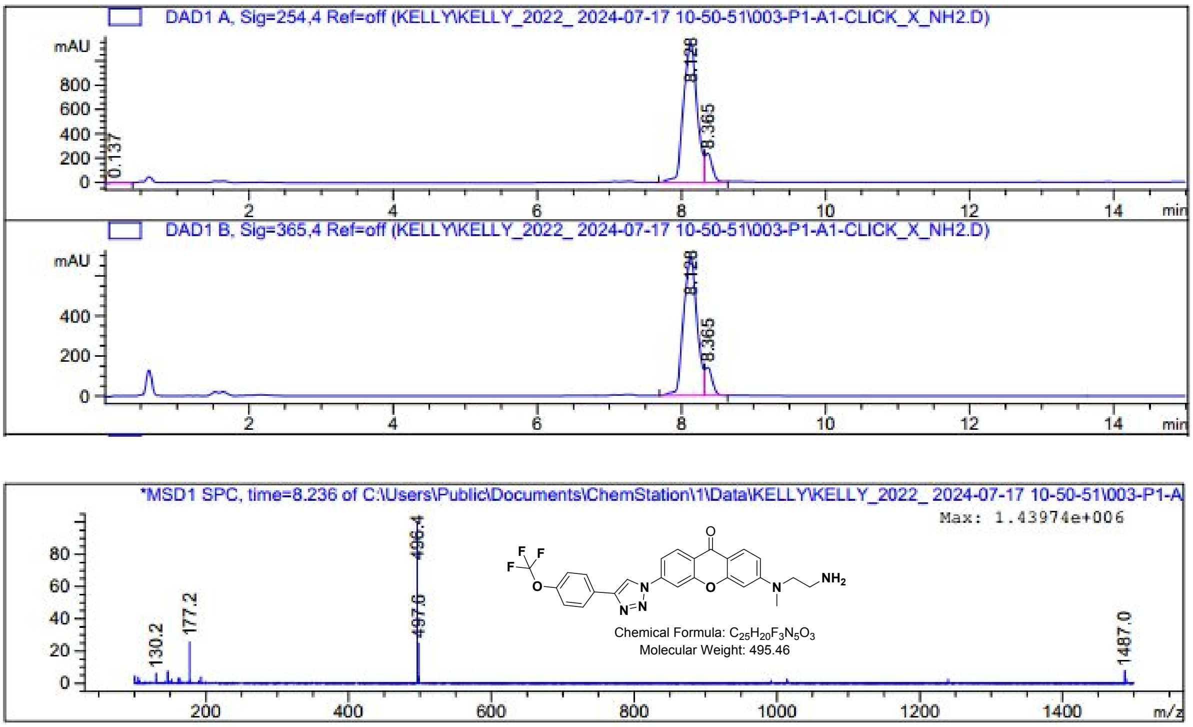

Figure S13. HPLC chromatography and mass spectrum of compound **14** (R^2 building block 24). Top: HPLC chromatography of compound **14** (R^2 building block 24). Absorbance wavelength: 254 and 365 nm (top to bottom). A: H_2O w 0.1% TFA, B: ACN w 0.1% TFA, gradient from 10%B to 99%B in 15 min; column: Agilent InfinityLab Poroshell 120 CS-C18 (2.1 x 50 mm, 2.7 µm particle size). Bottom: mass spectrum of compound **14** (R^2 building block 24) extracted from t_R = 8.24 min. Where t_R means retention time, and TFA is the abbreviation of trifluoroacetic acid.

General procedure for solid-phase Grignard reactions and cleavage (CROS compounds).

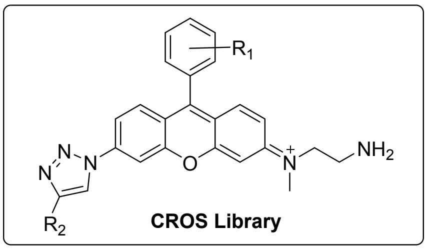

To 1 eq of resin (**14**) in THF in a 20 ml vial, 5 eq of Grignard reagent was added and heated at 65 °C for 48 hours. Then the resin was washed with DMF (5X), MeOH (5X), and DCM (5X). The product was then cleaved from the resin using 2% TFA in DCM (5ml for 15 mints) collected in a vial and evaporated to get the CROS Products.

General procedure for solid-phase reactions of CROS-AC & CROS-CA

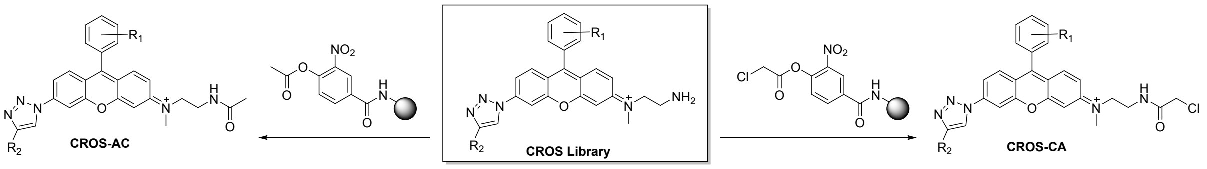

20mg of active ester resin was suspended in 400 µl of DCM/ACN (7:1). 1umol of each CX compound was also dissolved in 200 µl of same solvent and added to the resin shake it at room temperature. After 3 hour the solution was filtered and dried it in fume hood.

<u>Characterization of CROS-E24</u>

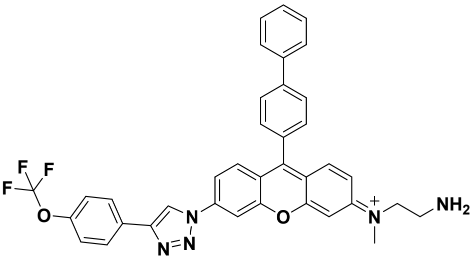

Chemical Formula: $C_{37}H_{29}F_3N_5O_2^+$
Exact Mass: 632.23
Molecular Weight: 632.67

CROS-E24

^{1}H NMR (600 MHz, MeOD-*d4*): δ (ppm) 8.98 (s, 1H), 8.01 (dd, 2H, J = 1.8, 6.7 Hz), 7.79 (d, 1H, J = 2.1 Hz), 7.59 (dd, 1H, J = 1.8, 8.6 Hz), 7.53-7.54 (m, 2H), 7.49 (d, 2H, J = 7.9 Hz), 7.42 (d, 2H, J = 7.9 Hz), 7.36-7.38 (m, 5H), 7.28 (t, J =7.4 Hz, 1H), 7.06 (d, 1H, J = 8.6 Hz), 6.67 (dd, 2H, J = 2.6, 11.5 Hz), 4.55 (s, 1H, NH_2), 3.63 (t, J = 6.6 Hz, 2H), 3.15 (t, J = 6.6 Hz, 2H), 3.02 (s, 3H). ^{13}C NMR (151 MHz, MeOD-*d4*): δ (ppm) 152.61, 150.34, 149.17, 148.03, 147.02, 140.62, 139.57, 137.19, 131.29, 130.16, 129.28, 128.50, 127.17, 127.02, 126.75, 126.58, 126.13, 124.31, 121.30, 119.40, 114.84, 109.84, 107.72, 98.62, 75.59, 49.81, 49.69, 37.71, 36.89. LC-MS (ESI) $[M]^+$: *m/z* calcd for $C_{37}H_{29}F_3N_5O_2$ 632.2, found 632.4. HR-MS (FAB) $[M]^+$: *m/z* calcd for 632.2268, found 632.2207 (Δm = -9.648 ppm).

NMR and LC-MS of CROS-E24

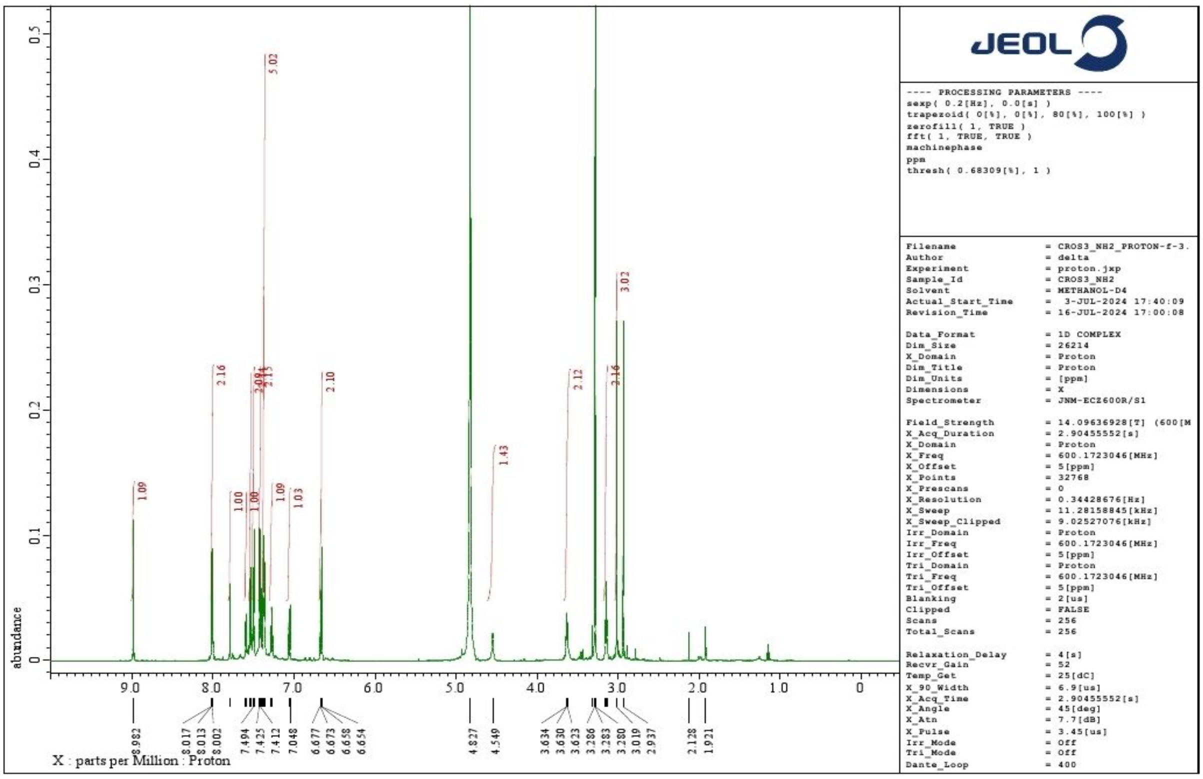

Figure S14. ^{1}H NMR of **CROS-E24** (600 MHz, MeOD-*d4*).

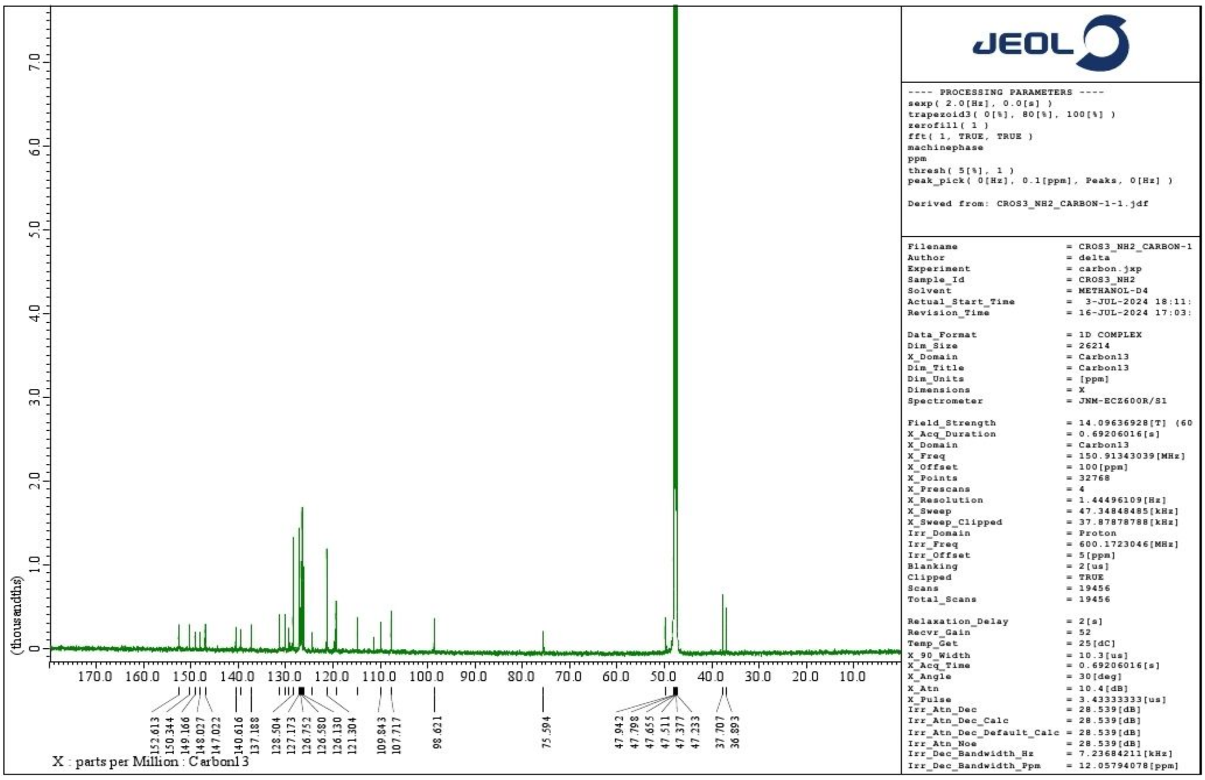

Figure S15. ^{13}C NMR of **CROS-E24** (151 MHz, MeOD-*d4*).

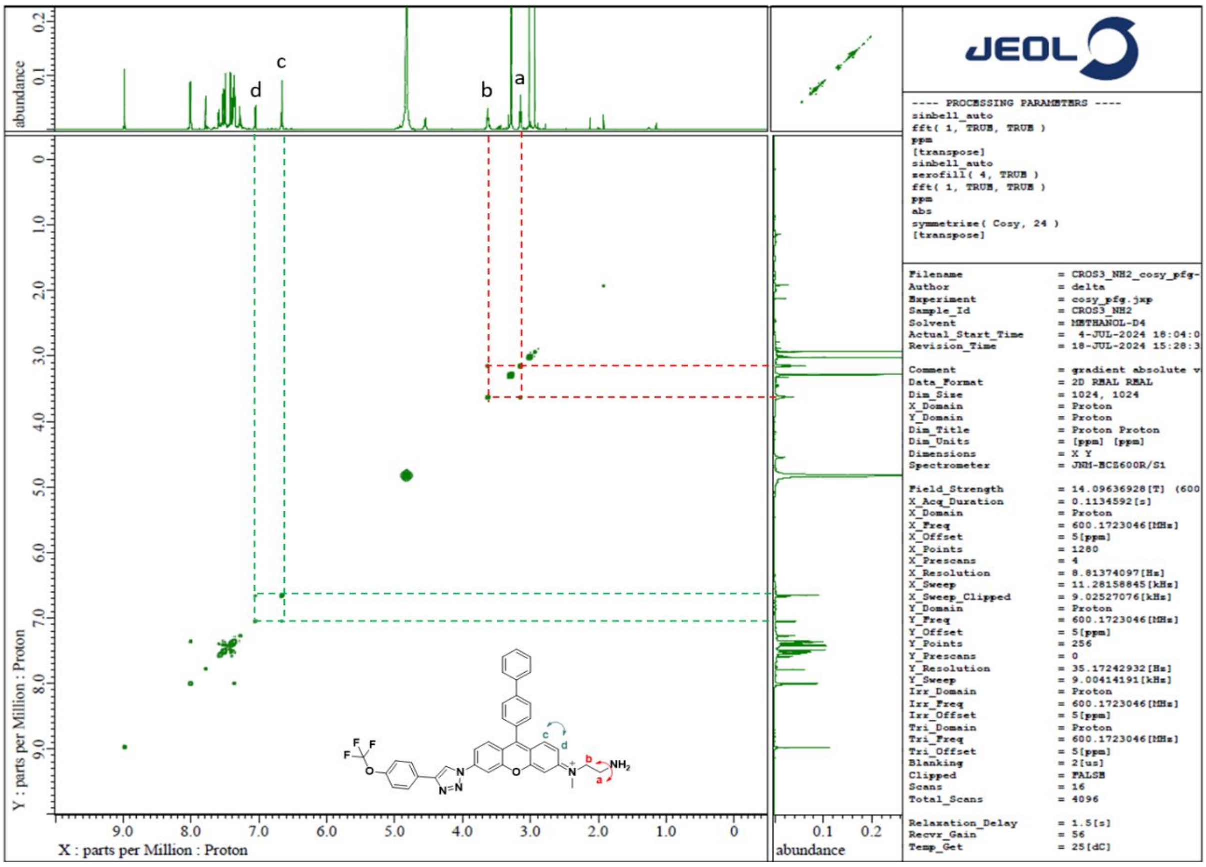

Figure S16. ^{1}H-^{1}H COSY of **CROS-E24** (600 MHz, MeOD-*d4*).

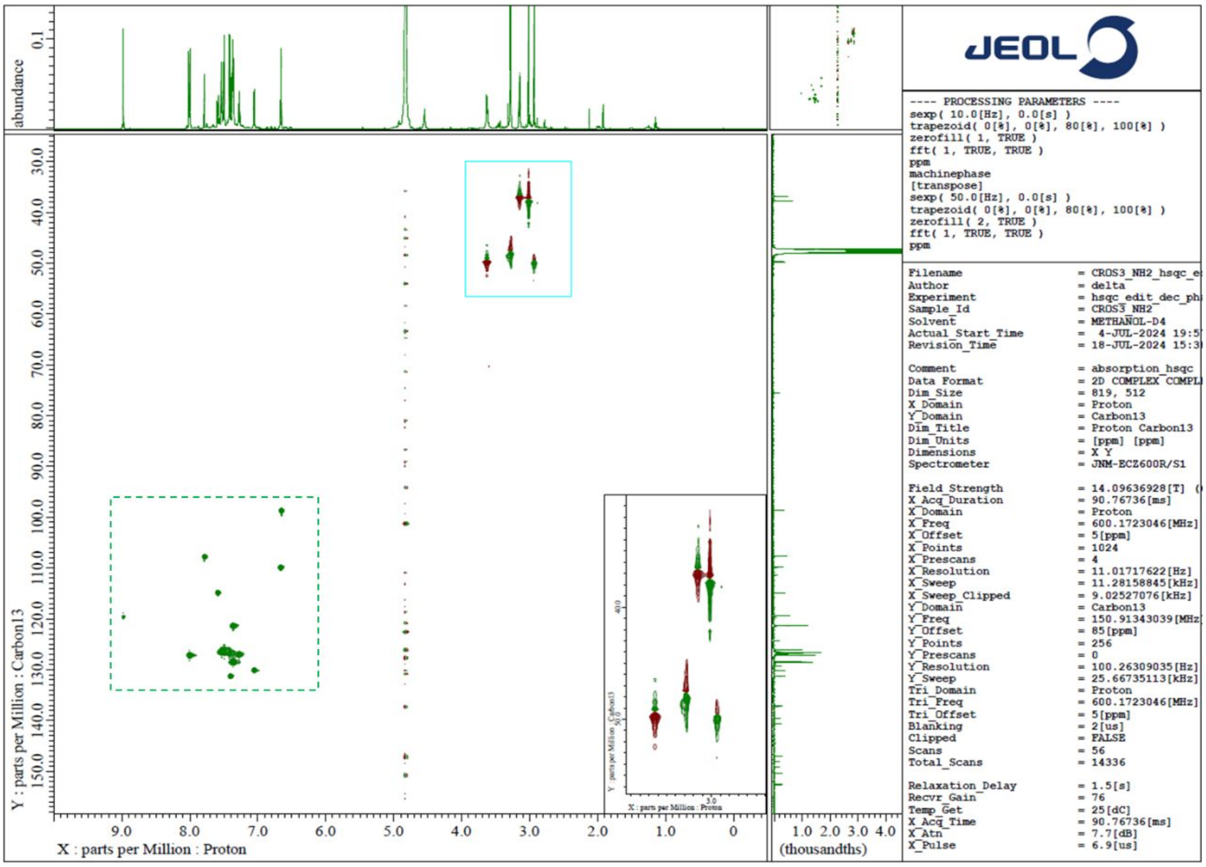

Figure S17. ^{1}H-^{13}C HSQC of **CROS-E24** (600 MHz for ^{1}H and 151 MHz for ^{13}C, MeOD-*d4*).

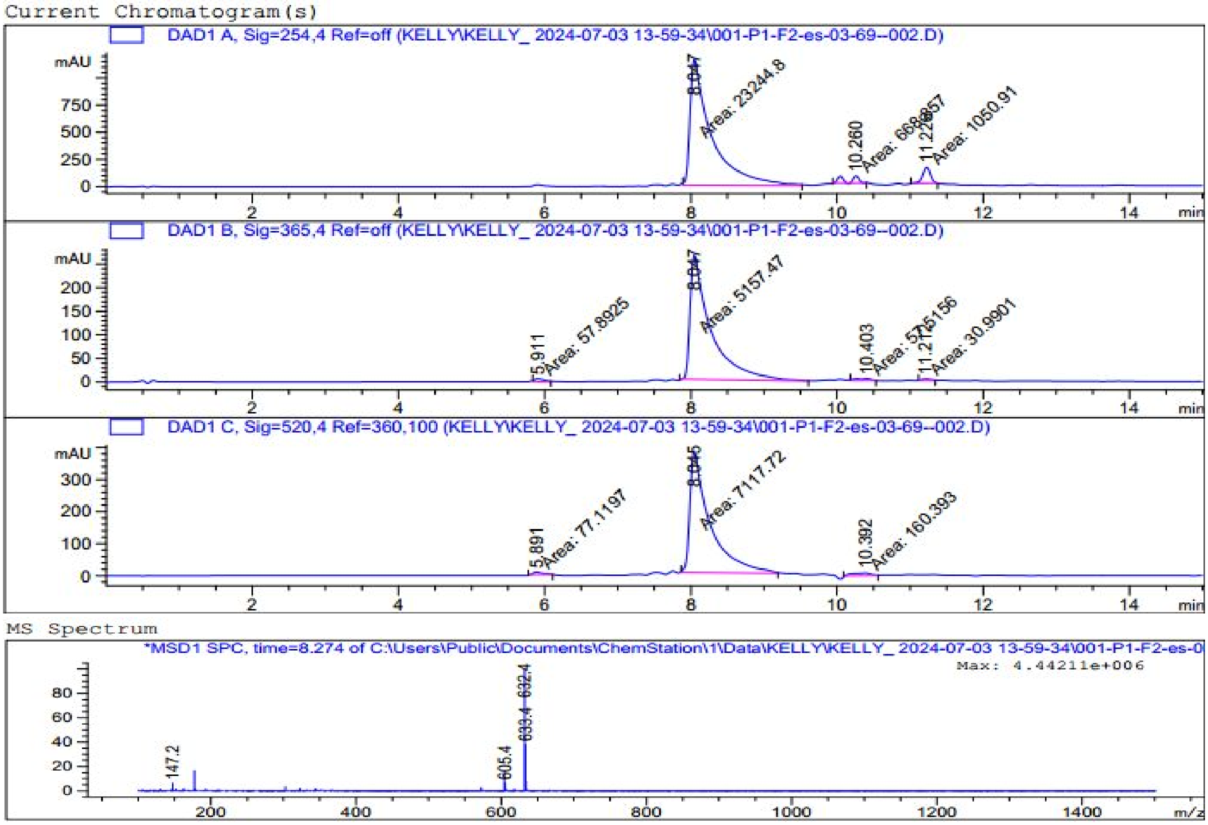

Figure S18. HPLC chromatography and mass spectrum of **CROS-E24**. Top: HPLC chromatography of **CROS-E24**. Absorbance wavelength: 254, 365, 520 nm. A: H_2O w 0.1% TFA, B: ACN w 0.1% TFA, gradient from 10%B to 99%B in 15 min; column: Agilent InfinityLab Poroshell 120 CS-C18 (2.1 x 50 mm, 2.7 µm particle size). Bottom: mass spectrum of **CROS-E24** extracted from t_R = 8.27 min. Where t_R means retention time, and TFA is the abbreviation of trifluoroacetic acid.

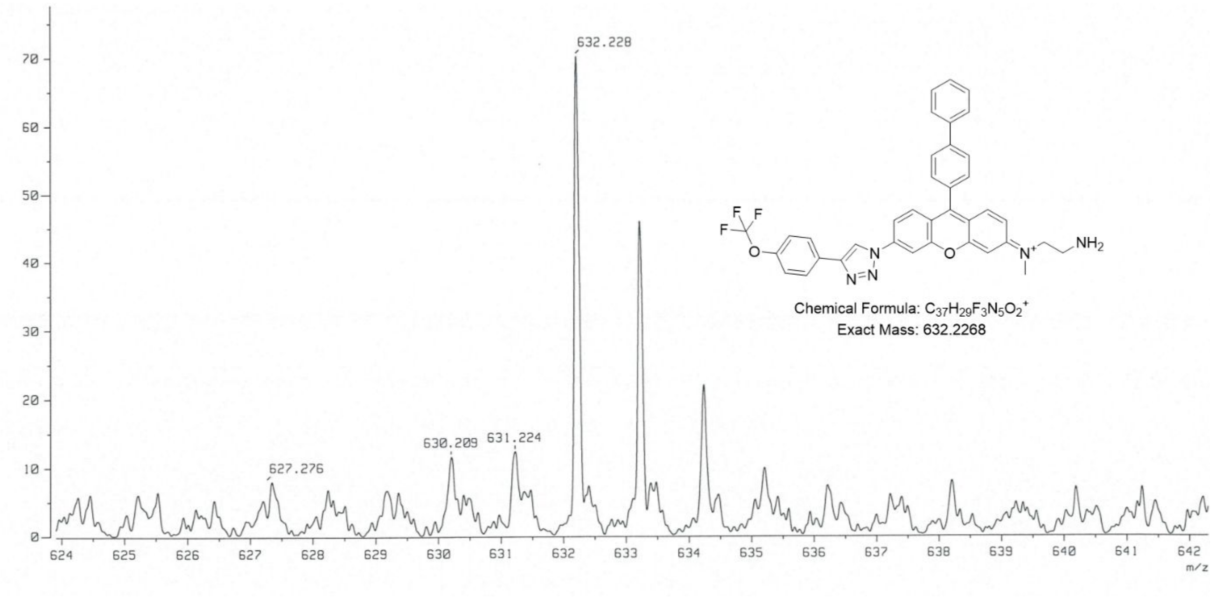

Figure S19. High resolution mass spectrum of **CROS-E24**.

Characterization of hLR

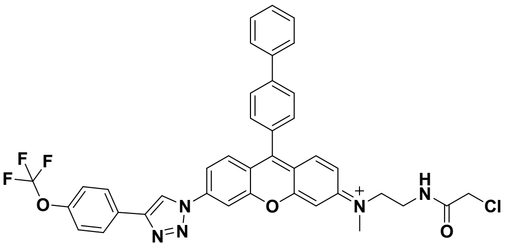

Chemical Formula: $C_{39}H_{30}ClF_3N_5O_3^+$
Exact Mass: 708.20
Molecular Weight: 709.15
hLR

^{1}H-NMR (600 MHz, MeOD-*d4*) δ (ppm) 8.94 (s, 1H), 7.99 (dd, J = 2.1, 6.6 Hz, 2H), 7.74 (dd, J = 2.1,6.6 Hz, 1H), 7.55 (dd, J = 2.1, 8.4 Hz, 1H), 7.52 (dd, J = 0.9, 8.4 Hz, 2H), 7.47 (d, J = 8.4 Hz, 2H), 7.40 (d, J = 8.4 Hz, 2H), 7.34-7.37 (m, 5H), 7.26 (t, J = 7.4 Hz, 1H), 6.95 (d, J = 9.0 Hz, 1H), 6.60 (dd, J = 2.8, 9.0 Hz, 1H), 6.54 (d, J = 2.4 Hz, 1H), 3.86 (s, 2H), 3.52 (d, J = 9.0 Hz, 2H), 3.42-3.43 (m, 2H), 2.97 (s, 3H). ^{13}C-NMR (151 MHz, MeOD-*d4*) δ (ppm) 168.17, 152.70, 152.63, 150.41, 149.14, 148.15, 146.96, 140.67, 139.45, 137.05, 131.23, 129.80, 129.29, 128.47, 127.16, 126.94, 126.79, 126.60, 126.10, 124.47, 121.28, 119.35, 114.65, 109.80, 109.26, 107.70, 97.53, 75.59, 50.42, 41.75, 37.29, 37.03. LC-MS (ESI) $[M]^+$: *m/z* calcd for $C_{39}H_{30}ClF_3N_5O_3$ 708.2, found 708.6.

NMR and LC-MS of hLR

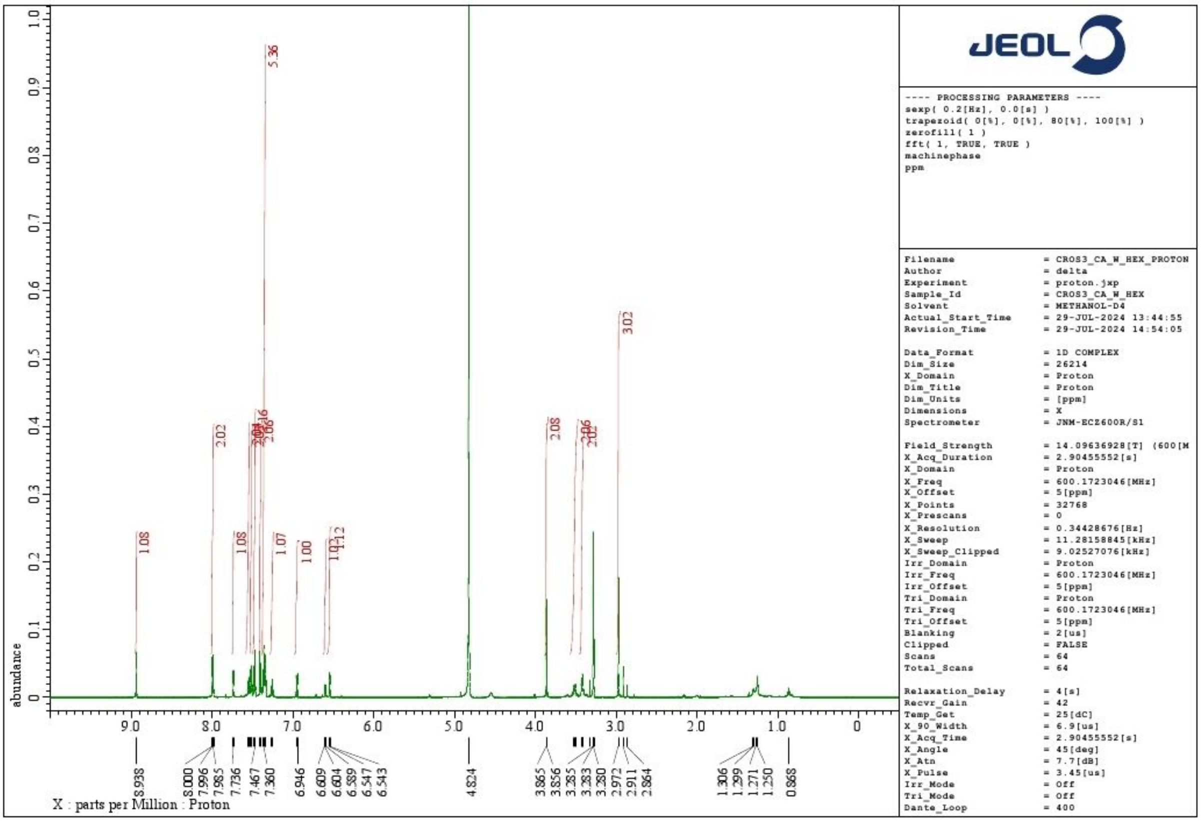

Figure S20. ^{1}H NMR for **hLR** (600 MHz, MeOD-*d4*).

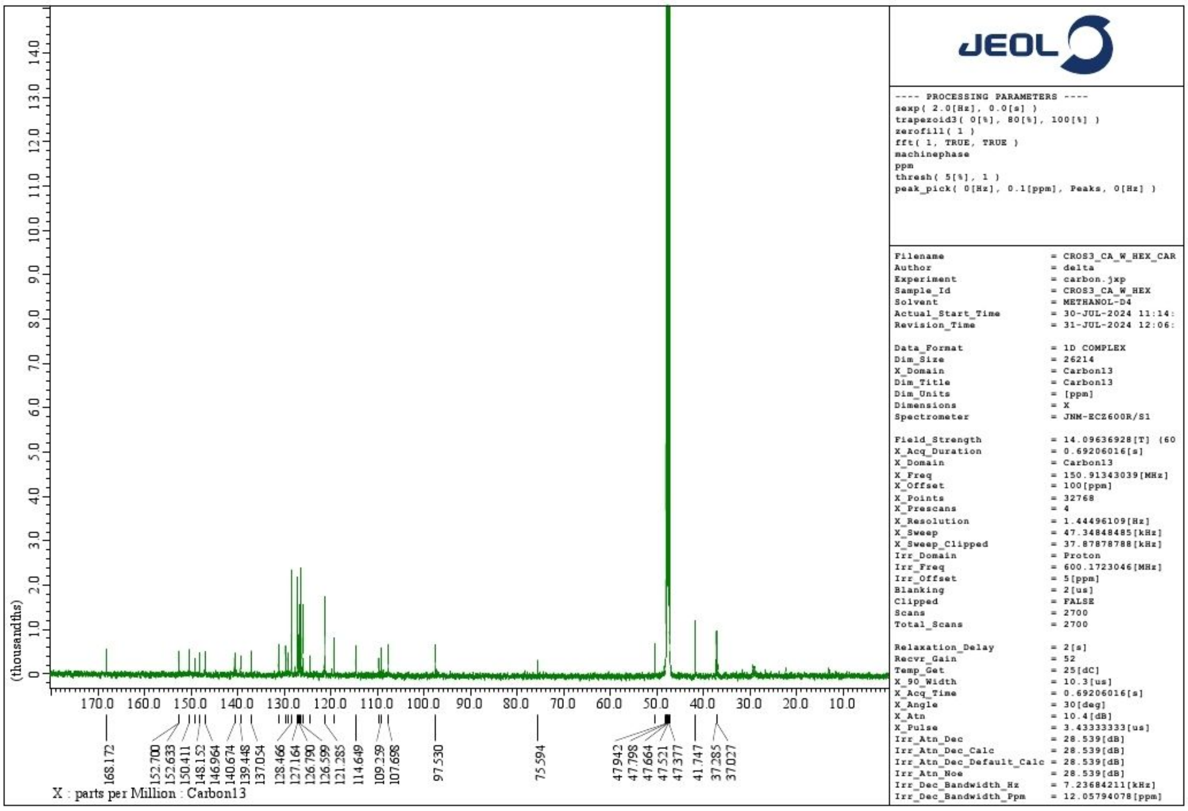

Figure S21. ^{13}C NMR of **hLR** (151 MHz, MeOD-*d4*).

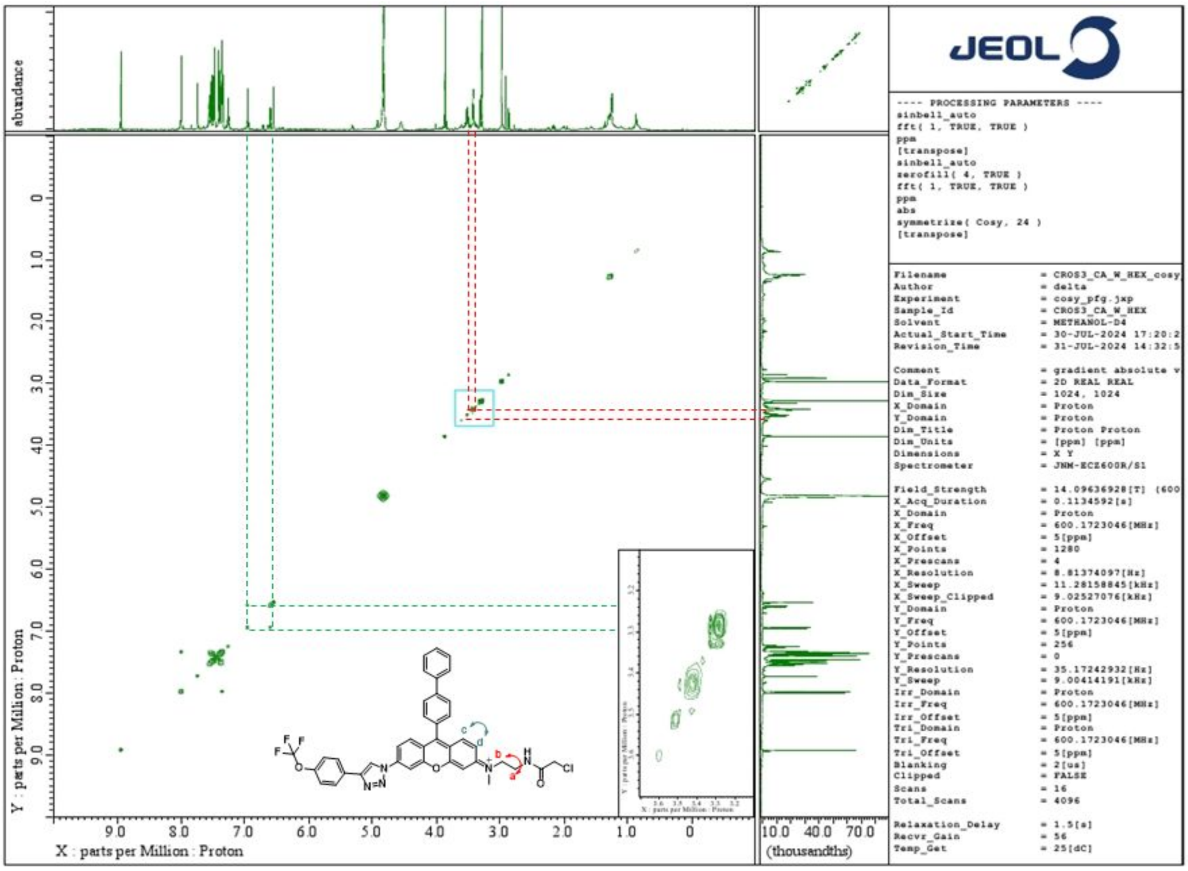

Figure S22. ^{1}H-^{1}H COSY of **hLR** (600 MHz, MeOD-*d4*).

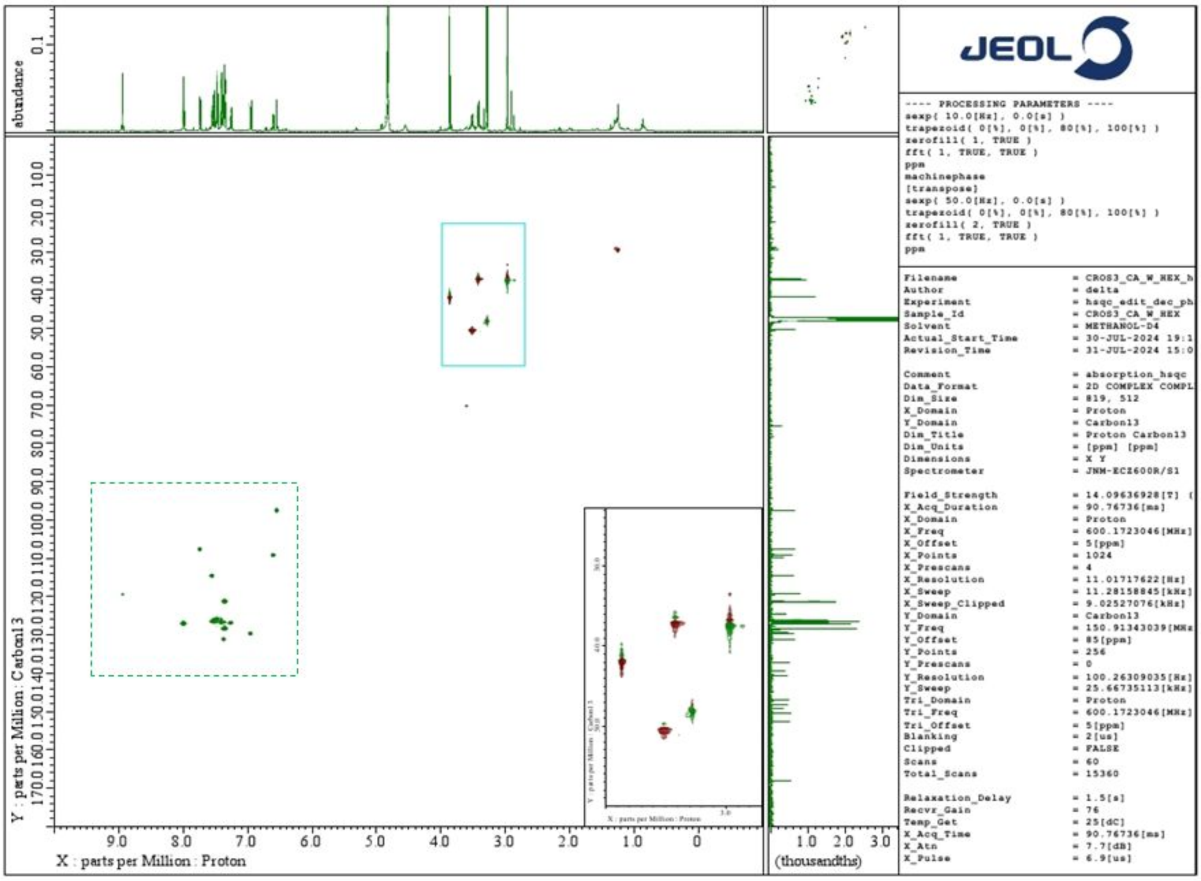

Figure S23. ^{1}H-^{13}C HSQC of **hLR** (600 MHz for ^{1}H and 151 MHz for ^{13}C, MeOD-*d4*).

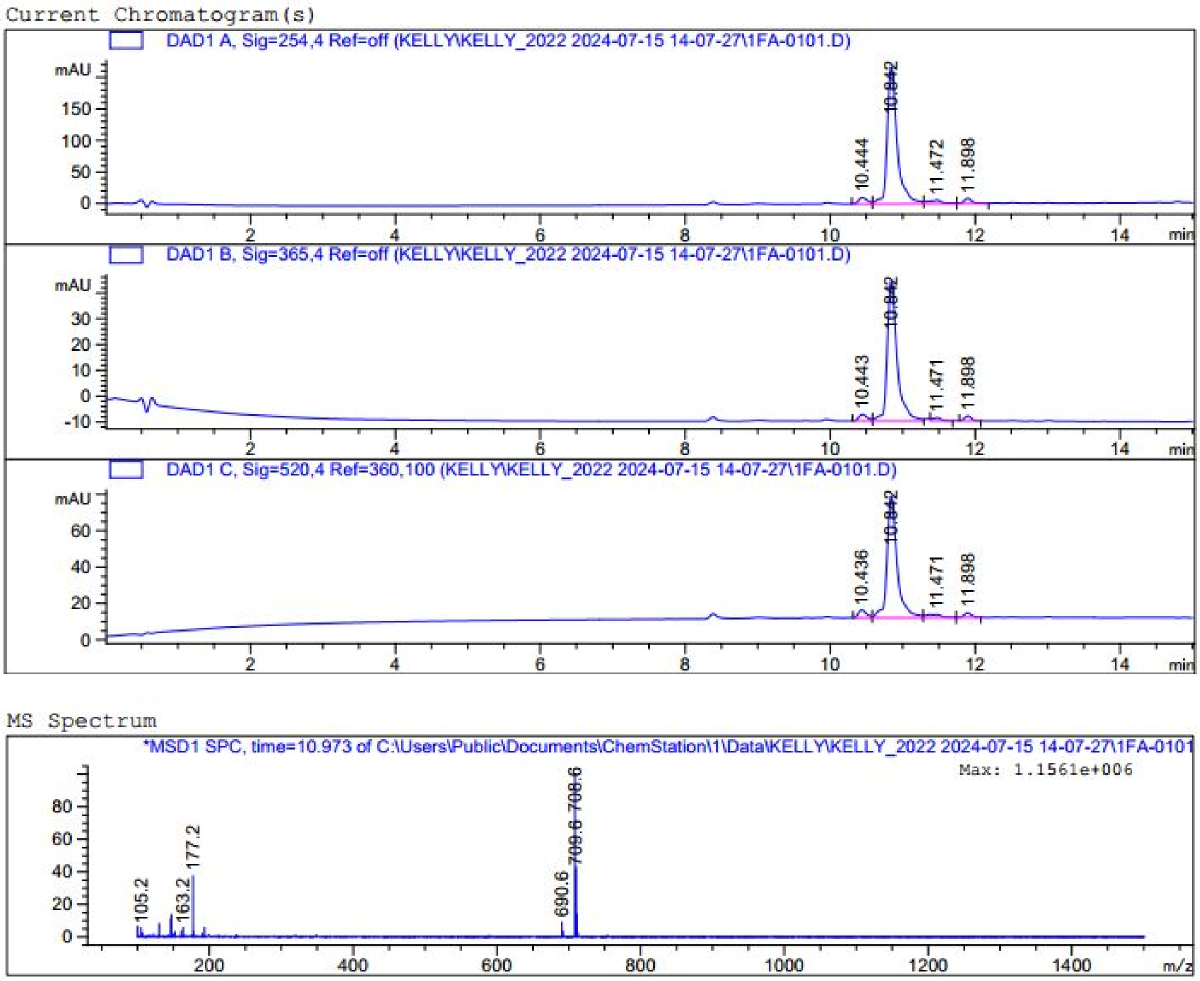

Figure S24. Mass spectrum and HPLC chromatography of **hLR**. Top: HPLC chromatography of **hLR**. Absorbance wavelength: 254, 365, 520 nm. A: H_2O w 0.1% TFA, B: ACN w 0.1% TFA, gradient from 10%B to 99%B in 15 min; column: Agilent InfinityLab Poroshell 120 CS-C18 (2.1 x 50 mm, 2.7 µm particle size). Bottom: mass spectrum of **hLR** extracted from t_R = 10.97 min. Where t_R means retention time, and TFA is the abbreviation of trifluoroacetic acid.

3. Optical properties of of cLG

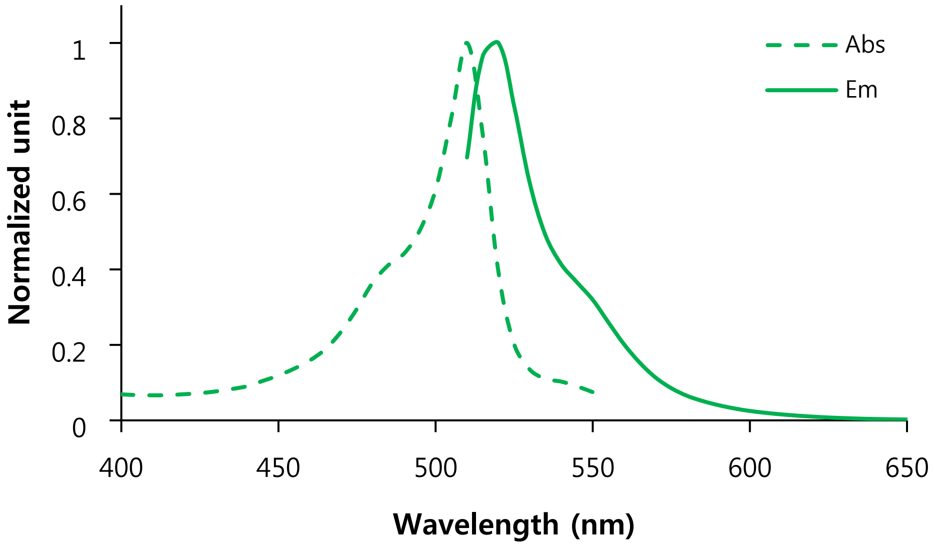

Figure S25. Absorbance and fluorescence spectra for **cLG**. Measurement condition: 10 μM **cLG** in DMSO and the emission spectrum was recorded excited at λ_{ex}= 500 nm.

Solvent	λ_{abs} (nm)	λ_{em} (nm)[a]	ε ($M^{-1}cm^{-1}$)	Φ_{fl}
DMSO	510	518	6.56 x 10^4	0.87
PBS (pH=7.4)	509	515	6.65 x 10^4	0.63

Table S1. Optical properties of **cLG**. [a] the emission wavelength was measured at λ_{ex}= 500 nm.

4. Optical properties of hLR

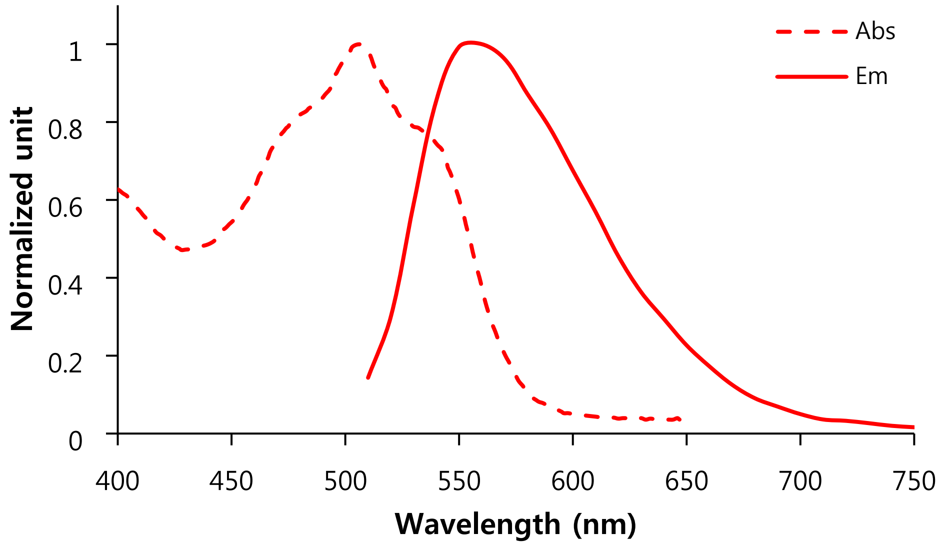

Figure S26. Absorbance and fluorescence spectra for **hLR**. Measurement condition: 10 μM **hLR** in MeOH and the emission spectrum was recorded excited at λ_{ex}= 500 nm.

Solvent	λ_{abs} (nm)	λ_{em} (nm)[a]	ε ($M^{-1}cm^{-1}$)	Φ_{fl}
MeOH	506	560	7.83 x 10^3	0.04
PBS (pH=7.4)	560	554	2.13 x 10^4	0.001

Table S2. Optical properties of **hLR**. [a] the emission wavelength was measured at λ_{ex}= 500 nm.

5. Cytotoxicity of cLG and hLR in cells

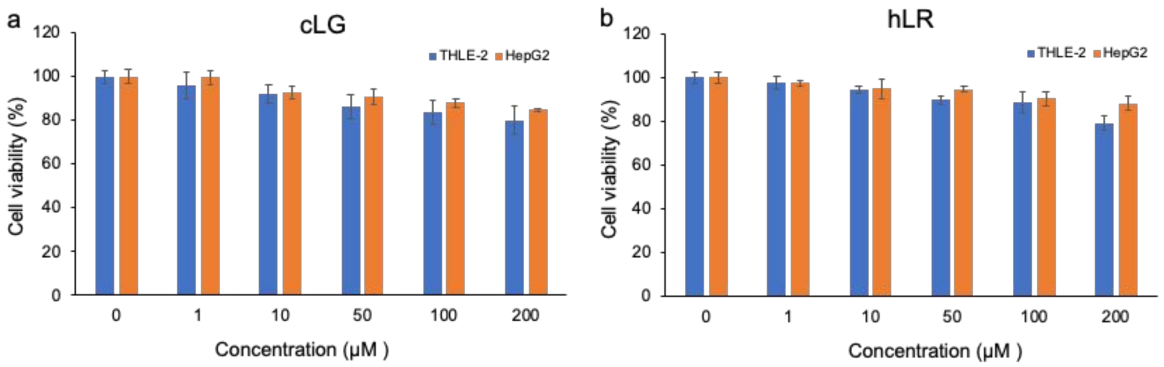

Figure S27. Toxicity of probe cLG (a) and hLR (b) on THLE-2 and HepG2 cells.

6. Cell selectivity test

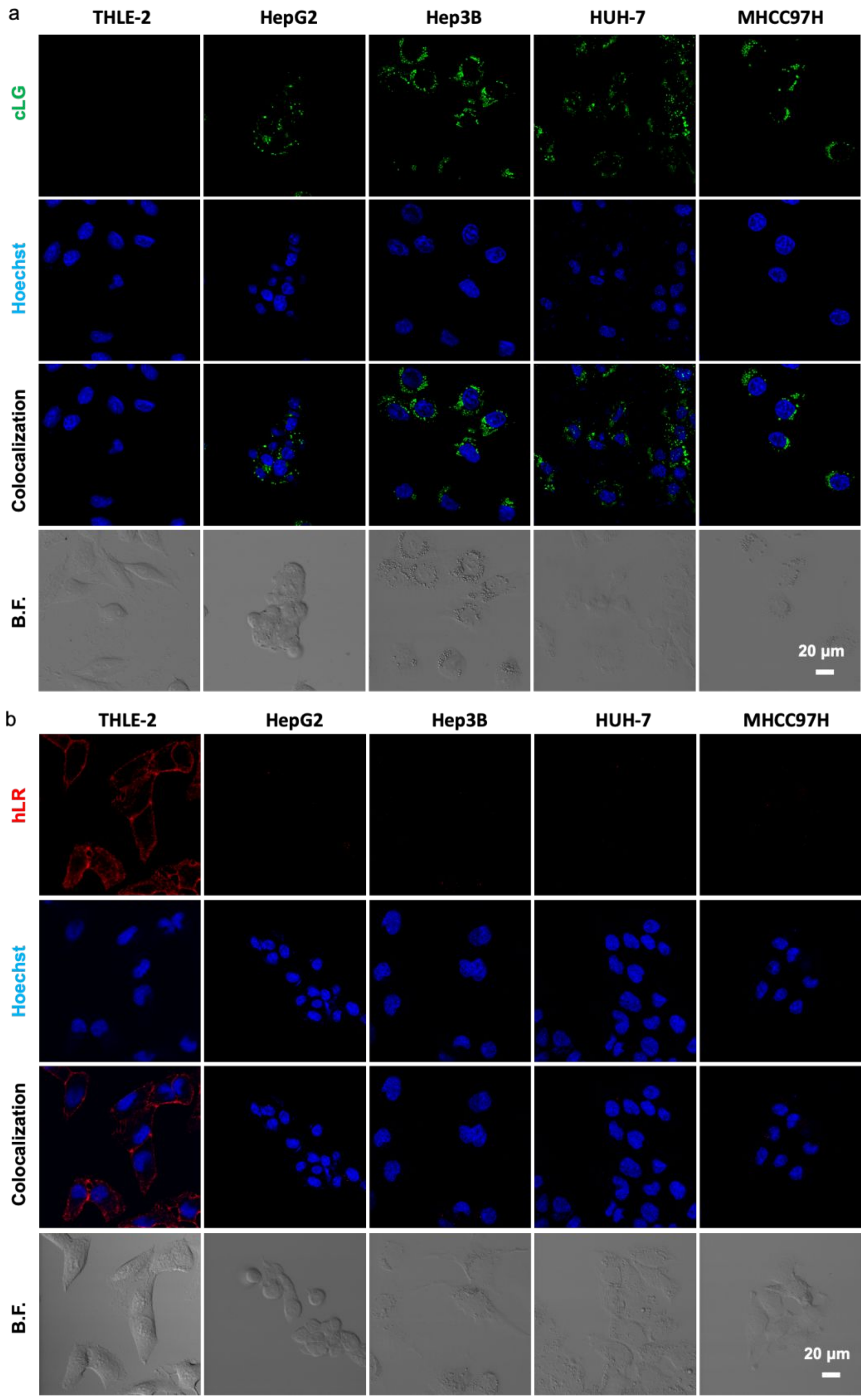

Figure S28. Fluorescent imaging of cLG/ hLR in different cell lines. Fluorescence images showed the intracellular fluorescent intensity of cells treated with cLG/ hLR (1 μM) and Hoechst (1 μg/mL) for 1 h and 10 min, respectively.

7. Cell localization

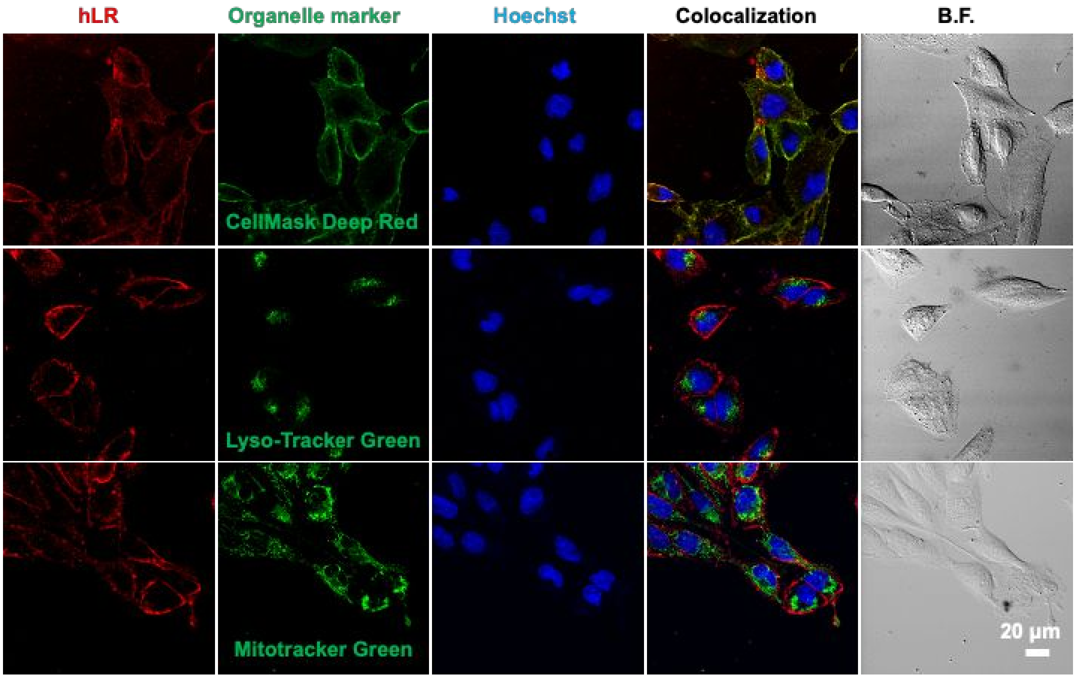

Figure S29. Localization of hLR in THLE-2 cells. Fluorescence images showed the intracellular fluorescent intensity of THLE-2 cells after treated with hLR (1 μM), Cellmask Deep Red (500 nM)/Lyso-Tracker (500 nM)/Mitotracker Green (200 nM) and Hoechst (1 μg/mL) for 1 h, 30 min and 10 min, respectively.

8. Mouse model of DEN-induced liver cancer

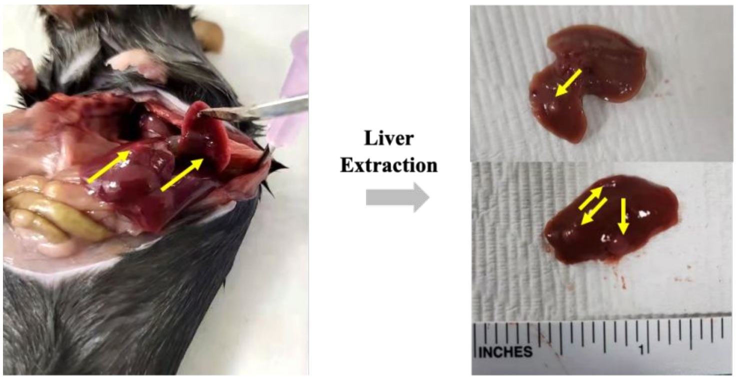

Figure S30. Establishment of a mouse model of liver cancer. Mice were killed 8 months after DEN administration. Yellow arrow indicates the tumor.

9. *Ex vivo* imaging of cLG/hLR

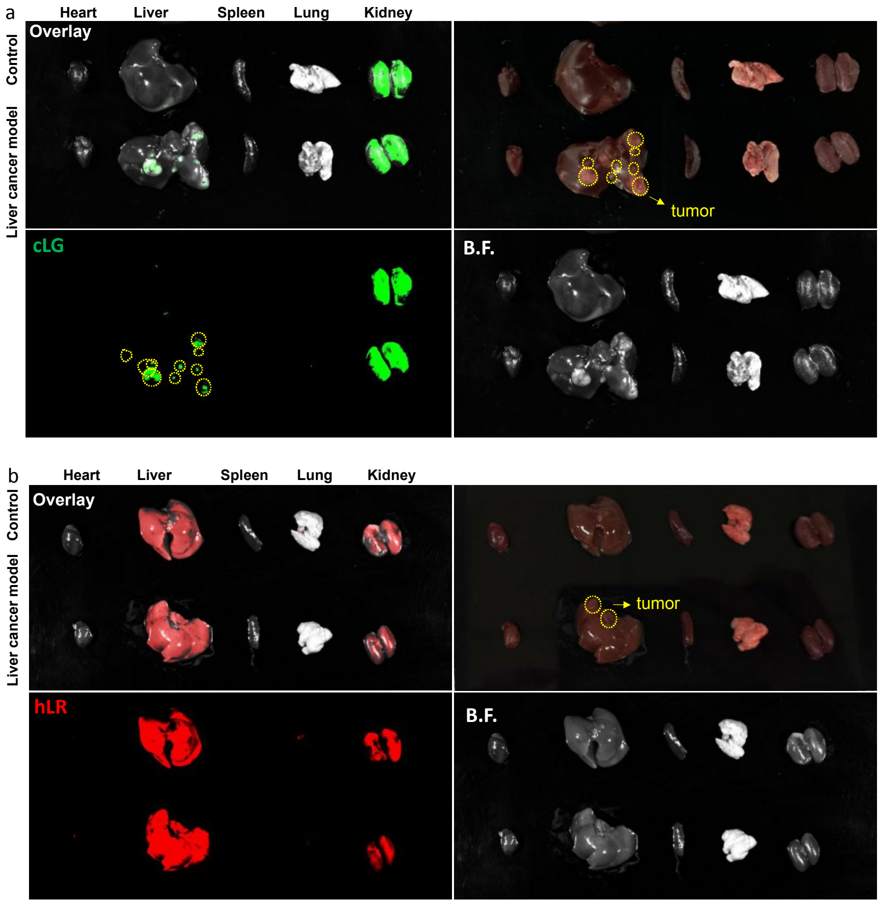

Figure S31. The *ex vivo* imaging of **cLG/hLR**.

10. Fluorescence enrichment of the cLG stained SLC-CRISPRa

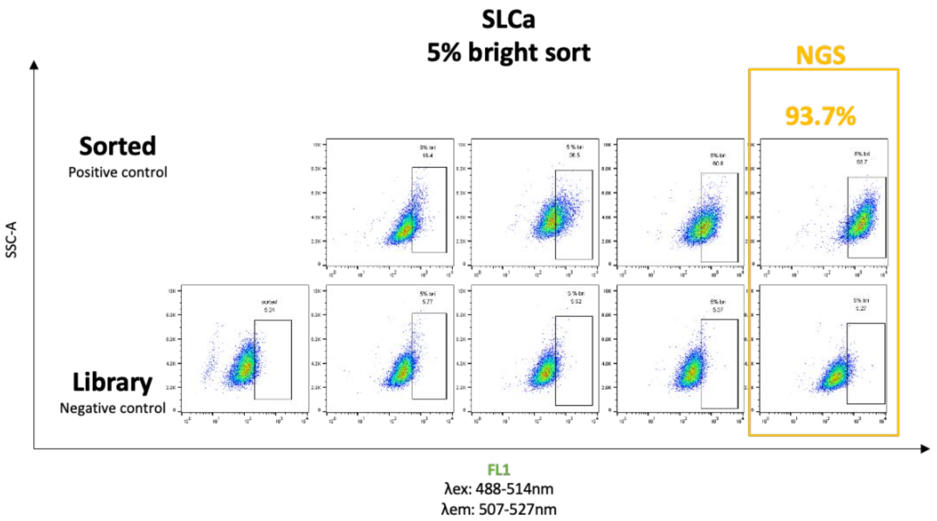

Figure S32. Fluorescence enrichment of the **cLG** stained SLC-CRISPRa. The enrichment process for the SLC-CRISPRa of **cLG** during 4th round. The cells were incubated with **cLG** (500 nM) for 30 min. Sorted, 5% bright population (black box).

11. mRNA expression of SMPD1

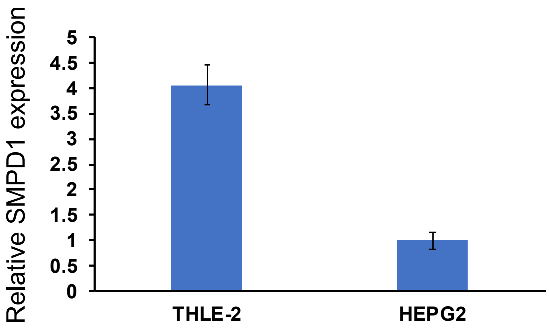

Figure S33. SMPD1 mRNA expression in THLE-2 and HepG2 cells evaluated by RT-PCR. Data pooled from three individual experiments. Data are analyzed with three samples over three independent experiments ($n = 3$).

12. References

1. Shirakami, Y.; Gottesman, M. E.; Blaner, W. S., Diethylnitrosamine-induced hepatocarcinogenesis is suppressed in lecithin:retinol acyltransferase-deficient mice primarily through retinoid actions immediately after carcinogen administration. *Carcinogenesis* **2012,** *33* (2), 268-274.

2. Lu, X.; Liao, B.; Sun, S.; Mao, Y.; Wu, Q.; Tian, R.; Tan. C.S.H., Scaled-Down Thermal Profiling and Coaggregation Analysis of the Proteome for Drug Target and Protein Interaction Analysis. *Anal. Chem.* **2023**, *95*, 13844−13854.

3. Ji, H.; Lu, X.; Zhao, S.; Wang, Q.; Liao, B.; Bauer, L. G.; Huber, K.V.M.; Luo, R.; Tian, R.; Tan. C.S.H., Target deconvolution with matrix-augmented pooling strategy reveals cell-specific drug-protein interactions. *Cell Chem. Bio.* **2023**, *30,* 1478−1487.

4. Würth, C.; Grabolle, M.; Pauli1, J.; Spieles, M.; Resch-Genger, U., Relative and absolute determination of fluorescence quantum yields of transparent samples. *Nat. Protoc.* **2013**, *8*, 1535-1550.

5. Gao, M. *et al.*, Neutrophil-Selective Fluorescent Probe Development through Metabolism-Oriented Live-Cell Distinction. *Angew. Chem. Int. Ed.* **2021**, *60*, 23743–23749.
